# Optical mapping of human embryonic stem cell-derived cardiomyocyte graft electrical activity in injured hearts

**DOI:** 10.1186/s13287-020-01919-w

**Published:** 2020-09-25

**Authors:** Dominic Filice, Wahiba Dhahri, Joell L. Solan, Paul D. Lampe, Erin Steele, Nikita Milani, Benjamin Van Biber, Wei-Zhong Zhu, Tamilla Sadikov Valdman, Rocco Romagnolo, José David Otero-Cruz, Kip D. Hauch, Matthew W. Kay, Narine Sarvazyan, Michael A. Laflamme

**Affiliations:** 1 Department of Bioengineering, University of Washington, Seattle, WA 98195 USA; 2 Institute for Stem Cell & Regenerative Medicine, University of Washington, Seattle, WA 98195 USA; 3 McEwen Stem Cell Institute, University Health Network, 101 College Street, Rm 3-908, Toronto, ON M5G 1L7 Canada; 4 Peter Munk Cardiac Centre, University Health Network, Toronto, ON M5G 2N2 Canada; 5 Fred Hutchinson Cancer Research Center, Seattle, WA 98109 USA; 6 Department of Biology, University of Washington, Seattle, WA 98195 USA; 7 Department of Pathology, University of Washington, Seattle, WA 98195 USA; 8 Department of Biomedical Engineering, G. Washington University, Washington, DC 20052 USA; 9 Department of Pharmacology & Physiology, G. Washington University, Washington, DC 20052 USA; 10 Department of Laboratory Medicine & Pathobiology, University of Toronto, Toronto, ON M5G 1L7 Canada

**Keywords:** Human embryonic stem cells, Cardiomyocyte, Cell transplantation, Optical mapping, Cardiac electrophysiology

## Abstract

**Background:**

Human embryonic stem cell-derived cardiomyocytes (hESC-CMs) show tremendous promise for cardiac regeneration, but the successful development of hESC-CM-based therapies requires improved tools to investigate their electrical behavior in recipient hearts. While optical voltage mapping is a powerful technique for studying myocardial electrical activity ex vivo, we have previously shown that intra-cardiac hESC-CM grafts are not labeled by conventional voltage-sensitive fluorescent dyes. We hypothesized that the water-soluble voltage-sensitive dye di-2-ANEPEQ would label engrafted hESC-CMs and thereby facilitate characterization of graft electrical function and integration.

**Methods:**

We developed and validated a novel optical voltage mapping strategy based on the simultaneous imaging of the calcium-sensitive fluorescent protein GCaMP3, a graft-autonomous reporter of graft activation, and optical action potentials (oAPs) derived from di-2-ANEPEQ, which labels both graft and host myocardium. Cardiomyocytes from three different GCaMP3+ hESC lines (H7, RUES2, or ESI-17) were transplanted into guinea pig models of subacute and chronic infarction, followed by optical mapping at 2 weeks post-transplantation.

**Results:**

Use of a water-soluble voltage-sensitive dye revealed pro-arrhythmic properties of GCaMP3+ hESC-CM grafts from all three lines including slow conduction velocity, incomplete host-graft coupling, and spatially heterogeneous patterns of activation that varied beat-to-beat. GCaMP3+ hESC-CMs from the RUES2 and ESI-17 lines both showed prolonged oAP durations both in vitro and in vivo. Although hESC-CMs partially remuscularize the injured hearts, histological evaluation revealed immature graft structure and impaired gap junction expression at this early timepoint.

**Conclusion:**

Simultaneous imaging of GCaMP3 and di-2-ANEPEQ allowed us to acquire the first unambiguously graft-derived oAPs from hESC-CM-engrafted hearts and yielded critical insights into their arrhythmogenic potential and line-to-line variation.

## Background

Human embryonic stem cells (hESCs) have a number of attractive properties for the repair of injured hearts, including tremendous capacity for in vitro expansion and the ability to differentiate into phenotypically unambiguous cardiomyocytes [1–8]. The transplantation of hESC-derived cardiomyocytes (hESC-CMs) has been shown to partially remuscularize injured hearts and to mediate beneficial effects on contractile function in mouse, rat, guinea pig, and non-human primate models of myocardial infarction [9–16]. Our group has shown that these cells form implants of human myocardium that are capable of partial electrical coupling and synchronous contraction with host myocardium during systole, a sine qua non of cardiac regeneration [13, 14]. However, we found that hESC-CM transplantation in porcine and non-human primate infarct models results in transient bouts of non-lethal ventricular tachycardia [16, 17], perhaps reflecting the immature phenotype of hESC-CMs and the significant electrophysiological mismatch between graft and host myocardium. While there are other remaining challenges related to scalability, graft cell immune rejection, and tumor formation, this phenomenon of graft-related arrhythmias has emerged as arguably the greatest barrier to the successful development of hESC-CM-based cardiac therapies. To address this issue, the field must develop new, more powerful tools to investigate the electrophysiological properties of hESC-CM-engrafted hearts and to test strategies to improve their electrical stability.

Our group has previously reported one approach to obtain useful insights into the electrical integration of hESC-CM grafts in normal and injured hearts [13]. For this work, we generated transgenic hESC-CMs that stably expressed the calcium-sensitive fluorescent protein GCaMP3 and exhibited robust fluorescent transients with each contraction cycle [18–20]. We transplanted these GCaMP3+ hESC-CMs into guinea pig [13, 14] and non-human primate hearts [16], which were then harvested at various timepoints post-transplantation and imaged ex vivo. By correlating the graft-autonomous GCaMP3 fluorescent signal with the host electrocardiogram (ECG), we were able to determine which GCaMP3+ hESC-CM grafts were electrically active and/or coupled with the ventricular myocardium of the recipient. In the guinea pig model, we found that all of the hESC-CM grafts in uninjured hearts were reliably 1:1 coupled with host myocardium, but outcomes were more complicated following transplantation into injured hearts. When we transplanted GCaMP3+ hESC-CMs in a subacute cardiac injury model (delivering cells at 10 days post-injury), we found that ~ 60% of engrafted hearts later showed some regions of 1:1 host-graft coupling [13]. The extent of electromechanical integration was reduced when cells were injected into a chronic injury model (at 28 days post-injury) with more established fibrosis and contractile dysfunction, and, in this case, only ~ 40% of recipient hearts showed 1:1 host-graft coupling [14].

While this approach based on GCaMP3 imaging alone has provided crucial insights into the integration of hESC-CM grafts, it does have a number of important limitations. First, GCaMP3 imaging yields no information as to electrical activation of host myocardium, precluding study of the complex spatiotemporal electrical interactions between graft and host tissue. Second, GCaMP3 senses the rise in intracellular calcium, which is obviously delayed relative to membrane depolarization. Moreover, because GCaMP3 is a particularly slow calcium sensor [19], GCaMP3 fluorescence transients substantially lag actual electrical activation in hESC-CMs. One attractive route to overcome these limitations would be to apply optical voltage mapping, a tool that has provided critical insights into normal cardiac propagation and mechanisms of arrhythmogenesis [21–28]. This technique involves labeling hearts with a fluorescent voltage-sensitive dye, imaging the resultant dye-derived optical action potentials (oAPs) with a high-speed camera or photodiode array, and post-processing of this imaging data to extract electrophysiological parameters of interest. Critical parameters including the pattern of electrical activation, action potential duration (APD), and tissue conduction velocity (CV) can all be acquired by optical mapping, making it an attractive tool for evaluating cell-engrafted hearts. There have been previous attempts to apply this technology to study the electrical activity of hESC-CM grafts after transplantation into uninjured hearts [29, 30]. However, our group has previously shown that hESC-CM grafts are not efficiently labeled by conventional voltage-sensitive dyes and that observed oAPs are actually derived from host rather than graft tissue [13].

We report here our efforts to overcome the above limitations using a novel approach that involves the simultaneous imaging of GCaMP3, the aforementioned genetically encoded calcium-sensitive fluorescent protein that functions as a graft-autonomous reporter of graft activation, and di-2-ANEPEQ, a water-soluble voltage dye that labels both host and graft tissue. This approach allows one to reliably distinguish between host- and graft-derived oAPs and to obtain previously unavailable electrophysiological measurements of hESC-CM graft tissue in injured hearts.

## Methods

### Generation of wild-type and GCaMP3+ hESC-CMs

We created transgenic H7, ESI-17, and RUES2 hESC lines that stably expressed GCaMP3 [18] via the targeted insertion of a previously reported expression cassette into the adeno-associated virus integration site 1 (AAVS1) “safe harbor” locus [13, 14]. In the case of H7 and RUES2 hESCs, this cassette was knocked-in by zinc finger nuclease-mediated transgenesis [31], while ESI-17 hESCs were equivalently targeted by CRISPR/Cas9-mediated gene-editing [32]. Wild-type (WT) and GCaMP3+ hESCs were then expanded and differentiated into cardiomyocytes via a previously reported guided differentiation protocol based on the sequential delivery of the growth factors activin A and bone morphogenetic protein-4 [9, 13, 33, 34]. By these methods, spontaneously beating cardiomyocytes were typically observed on or before day 10. On day 19, hESC-CMs were transiently heat-shocked with 42 °C medium to improve their survival post-transplantation [35]. On day 20, hESC-CMs were harvested enzymatically and cryopreserved as previously described [33]. Cardiomyocytes > 83% purity were generated with this protocol as estimated by flow cytometry for cardiac troponin T.

All hESC experiments were conducted with the approval of either the University of Washington ESC Research Oversight Committee or the Canadian Institutes of Health Research (CIHR) Stem Cell Oversight Committee (SCOC). Note that, while experiments were initiated with the RUES2 hESC line when the laboratory was at the University of Washington, all work with this line had to be discontinued upon relocation to our present institution (RUES2 hESCs are not included in the CIHR SCOC registry of lines approved for use in Canada).

### Spectral analysis of GCaMP3 and di-2-ANEPEQ in hESC-CMs

Spectrofluorimetry and spectral confocal microscopy were used to define the excitation and emission spectra of GCaMP3 and di-2-ANEPEQ (Invitrogen, Carlsbad, CA, USA) in intact cardiomyocytes. WT and GCaMP3+ hESC-CMs were stained by incubation with di-2-ANEPEQ (5 μM) at 37 °C for 10 min, spun down, switched to dye-free buffer, and used immediately. For spectrofluorimetry experiments, we employed a Spectra Max M2 microplate reader (Molecular Devices, Sunnyvale, CA, USA) and 96-well plates loaded with 2 × 10^6^ hESC-CMs per well. Absorbance was measured from 350 to 700 nm in 5 nm steps, while emission was determined from 500 to 800 nm in 5 nm steps following excitation at 480 nm (with a 495 nm cutoff filter). Confocal experiments were performed using a Zeiss LSM510-Meta confocal microscope (Carl Zeiss GmbH, Gottingen, Germany) operated in lambda-scanning mode, with excitation set to 488 nm and emission detected from 502 to 748 nm in 11 nm steps. We also simultaneously recorded GCaMP3 and di-2-ANEPEQ fluorescence transients by confocal microscopy in time-series mode using 505–530 nm and 650–754 nm bandpass filters, respectively.

### Cardiac injury and hESC-CM transplantation

All animal procedures were conducted with the approval of the local institutional animal care committees of either the University of Washington or University Health Network (Toronto) in compliance with corresponding national guidelines. We have previously described in detail our methods for the cardiac cryoinjury procedure, intra-cardiac cell injection, and subsequent harvesting of the heart for ex vivo imaging [13, 14, 36]. In brief, 650–700 g male Hartley guinea pigs were anesthetized with ketamine-xylazine induction, intubated, mechanically ventilated, and maintained with 1.5% isoflurane anesthesia. A thoracotomy was performed, and cardiac cryoinjury was induced by applying an 8-mm diameter, liquid-nitrogen-cooled aluminum probe to the left ventricular free wall four times for 30 s each. At either 10 days (subacute model) or 28 days (chronic model) post-injury, a repeat thoracotomy was performed, and the heart was directly injected with 1 × 10^8^ GCaMP3+ hESC-CMs. Cells were delivered in a pro-survival cocktail of factors that we have previously shown enhances graft retention and survival [9]. To prevent immune rejection of the graft cells, we treated the recipient animals with a regimen of cyclosporine (SQ, 15 mg/kg/day × 7 days, followed by 7.5 mg/kg/day maintenance thereafter) and methylprednisolone (IP, 2 mg/kg/day), starting 2 days prior to cell injection and continuing until the heart was harvested at euthanasia.

### Simultaneous GCaMP3 and di-2-ANEPEQ imaging of hearts ex vivo

Cryoinjured hearts with GCaMP3+ hESC-CM grafts were harvested at 2 weeks post-transplantation and immediately mounted on a modified Langendorff apparatus perfused with the following buffer (in mM): 25.0 NaHC0_3_, 1.2 MgSO_4_, 4.7 KCl, 118.0 NaCl, 1.2 KH_2_PO_4_, 11.0 glucose, 1.0 Na-pyruvate and 1.8 CaCl_2_, bubbled with 95% O_2_/5% CO_2_, pH adjusted to 7.35, and warmed to 37 °C. To arrest motion during ex vivo imaging, the perfusate was supplemented with blebbistatin (10 μM, Cayman Chemical, Ann Arbor, MI, USA). To label hearts with the conventional lipophilic voltage dye RH237, hearts were either perfused with RH237-containing buffer (40 μM) for 30 min or bolus-loaded with RH237 (80 μM in 5 ml volume) over 5 min. Identical results were obtained with either method. To label hearts with the water-soluble voltage dye di-2-ANEPEQ, the latter was added to the perfusion buffer (20 μM concentration) and applied via continuous recirculation loop. Supplementary Fig. S1A provides an overview of the experimental protocol used to image hearts ex vivo with di-2-ANEPEQ. First, hearts were rapidly excised, mounted ex vivo on a modified Langendorff apparatus, and allowed to stabilize electrically (~ 10 min). Next, hearts were treated with blebbistatin in the perfusate to arrest motion (~ 10 min) and baseline recordings were made (“baseline,” ~ 10 min). Then, recordings were made while buffer containing both di-2-ANEPEQ and blebbistatin was recirculated through the heart (“di-2-ANEPEQ loading,” ~ 30 min), as well as after a switch back to dye-free but blebbistatin-containing buffer (“di-2-ANEPEQ washout,” ~ 30 min). During all periods, recordings were made under either sinus rhythm or paced conditions.

Supplementary Fig. S1B-C provides a detailed schematic of the imaging system used to acquire GCaMP3 and voltage dye (RH237 or di-2-ANEPEQ) signals. In brief, epicardial fluorescent transients were acquired using either a modified epifluorescence dissecting microscope (Nikon SMZ1000, Kawasaki, Kanagawa, Japan) outfitted with a × 0.5 objective (N.A. 0.05, W.D. 123 mm) or directly through a dichroic system (Photometrics DC2 dual-channel splitter, Tucson, AZ, USA) outfitted with a collimating lens (25 mm, Navitar, N.A. 0.05, W.D. 10 mm). Excitation light was provided by an external mercury lamp (EXFO X-Cite 120 W, Mississauga, Ontario, Canada) filtered to 450–490 nm. Fluorescence emission was imaged through the DC2 outfitted with a 565 nm dichroic mirror to separate GCaMP3 and voltage dye signals. The GCaMP3 emission signal (“green” channel) was bandpass-filtered to 500–530 nm, while the voltage dye signal (“red” channel) was longpass-filtered at 716+ nm for RH237 or 650+ nm for di-2-ANEPEQ. These signals were simultaneously detected by two high-speed, high-sensitivity EM-CCD cameras, either a pair of Andor iXon 860 EM-CCDs (Andor, Belfast, UK) or a pair of Evolve 128 EM-CCDs (Photometrics, Tucson, AZ, USA). Both cameras have an identical CCD60 sensor with a 128 × 128 imaging array, so are essentially interchangeable. The field of view (FOV) with these optics was 2.3 × 2.3 cm.

During these ex vivo imaging experiments, a pseudo-ECG was acquired using a PowerLab 430 Data Acquisition System (Model ML866) outfitted with a bioamplifier (Model ML136, ADInstruments, Colorado Springs, CO, USA), and the resultant signals were aligned with camera outputs via Labchart software. For recordings obtained under paced conditions, we used the PowerLab 430 system connected to a pencil-point concentric electrode (325 μm outer diameter stainless steel, 125 μm diameter inner iridium, FHC, Bowdoin, ME, USA) placed into the LV apex. For a subset of experiments, we obtained simultaneous intracellular voltage recordings via sharp electrodes, using methods modified from Omichi et al. [37]. For this, we impaled host or graft myocardium with pure iridium-tipped (1–2 μm diameter, 5 × 10^6^ ohm) electrodes coated with parylene-C insulation (World Precision Instruments, Shanghai, China). The resultant signals were amplified using a high-impedance intracellular electrometer with variable-capacity neutralization (Warner Instruments IE-251A intracellular electrometer, Hamden, CT, USA) and then fed into the PowerLab system.

### GCaMP3 and di-2-ANEPEQ imaging analysis

Custom Matlab (MathWorks) scripts were developed to analyze imaging data. In brief, time-synchronized fluorescent images and ECG recordings (from Andor Solis or Metamorph and LabChart software packages, respectively) were imported into the Matlab environment, and the former were subjected to the following sequence of initial processing steps. First, the images were masked to exclude signals outside the heart, then spatial, low pass frequency, and temporal filters were applied to improve signal quality. The results of filtering were then assessed at the single pixel level before removing signal drift. Next, the data was inverted for voltage dye-signals (which exhibit a decrease in fluorescence intensity upon tissue depolarization through the emission filters described), and a pseudocolored movie of activation based on the *z*-score of each pixel through time played to screen to facilitate identification of active graft regions. Next, we manually selected regions of interest (ROIs) and plotted their mean fluorescence intensity over time (with signals normalized to that of the region with the largest range in hearts with multiple ROIs). Activation maps of tissue were created by determining for each pixel the timepoint associated with the maximal rate of change in fluorescence activity between the initiation and peak of a single activation [38]. To aid in this determination, further drift removal, normalization, and careful utilization of a Savitzky-Golay filter were performed. Conduction velocity (CV) vectors were then determined from activation maps, given the known FOV. Optical action potential durations (oAPDs) and cycle lengths were measured in Matlab software by selecting a 10 × 10 pixel ROI and manually determining the start and end of each action potential (AP) from the region’s mean fluorescence intensity through time. The oAPD for a single graft ROI was then defined as the mean of 30 measured oAPDs. To rate-correct graft-derived oAPDs, we applied Fridericia’s formula [39], whereby the corrected oAPD equals the measured oAPD divided by the cube root of the measured cycle length.

### Imaging of hESC-CMs and hESC-CM aggregates in vitro

oAPs were recorded from WT and GCaMP3+ hESC-CMs in vitro as both single cells and ~ 300 μm diameter aggregates. For the single-cell recordings, hESC-CMs were plated onto gelatin-coated 23-mm glass-bottom fluorodishes (WPI, Sarasota, FL, USA). After 4–5 days, the cells were loaded with di-2-ANEPEQ (40 μM) for 5 min at 37 °C, then were transferred to dye-free buffer at 37 °C. Images were acquired using a × 20 objective and an Olympus IX-7 inverted microscope outfitted with an external mercury lamp (EXFO X-Cite 120 W, Mississauga, Ontario, Canada), filtered to 450–490 nm, and the same emission light path and EM-CCD cameras as described above. To form the aggregates, 3.6 × 10^6^ hESC-CMs were aliquoted per well into the commercially available AggreWell 800 system (StemCell Technologies, Vancouver, British Columbia, Canada) as per the manufacturer’s recommendations. Eight days later, the formed aggregates were loaded with di-2-ANEPEQ (20 μM) for 5 min at 37 °C, then dispersed and transferred into 23-mm fluorodishes for imaging at 37 °C. hESC-CM aggregates were imaged using the modified epifluorescence dissecting microscope and dual EM-CCD system described above for ex vivo epicardial imaging. Both the single-cell and aggregate preparations were field-stimulated at 1 Hz using custom-made parallel 0.25 mm diameter silver wire electrodes and the PowerLab system (with pulse duration set at 5 ms and voltage at 10 V). Analysis of in vitro optical recordings was performed using custom Matlab scripts as described above. The oAPD from each single cell or aggregate was defined as the mean of > 4 measured oAPDs, and reported results reflect at least > 12 recordings obtained from at least 3 differentiation runs per cell line.

### Histological analysis of GCaMP3+ hESC-CM-engrafted hearts

Histological endpoints were obtained using methods previously detailed by our group [9, 13, 40]. In brief, hearts were evaluated by routine histochemical stains (hematoxylin-eosin, picrosirius red, Masson’s trichrome), brightfield immunocytochemistry and/or immunofluorescence as previously reported [13, 17]. For immunostaining, we used primary antibodies against GFP (rabbit polyclonal), N-cadherin (mouse monoclonal), connexin-43 (Cx43) (obtained from A. Boynton), β-myosin heavy chain (clone A4.951), and the human specific nuclei marker Ku80 (Cell Signaling, Danvers, MA, USA), followed by detection with species-specific biotinylated (Vector Labs, Burlingame, CA, USA) or fluorescent (Life Sciences, Farmingdale, NY, USA) secondary antibodies.

Host-graft contact was determined using histological sections immunostained for GFP and counterstained with picrosirius red. Graft expression of Cx43 and cadherin expression was assessed by confocal immunofluorescence using a Zeiss LSM 780 NLO confocal microscope (Jena, Germany). All measurements were performed by an observer blinded to hESC line and experimental condition.

### Statistics

GraphPad Prism (GraphPad Software, La Jolla, California, USA) was used to perform all statistical analyses. All data groups were first checked for normality by submission to the D’Agostino-Pearson omnibus test, and parametric data is presented as mean ± standard error of the mean. Two-sample comparisons were made using a two-tailed Student’s *t* test, paired or unpaired as appropriate with Welch’s correction for unequal variances as necessary. Multiple comparisons were made using one-way ANOVA with Tukey’s multiple comparison test correction. A *p* value less than 0.05 was considered significant, and asterisks (*) used in figures indicate a significant difference between experimental groups.

## Results

### Conventional, lipophilic voltage-sensitive dyes do not label hESC-CM grafts, but the water-soluble dye di-2-ANEPEQ does label graft tissue

**Fig. 1:**
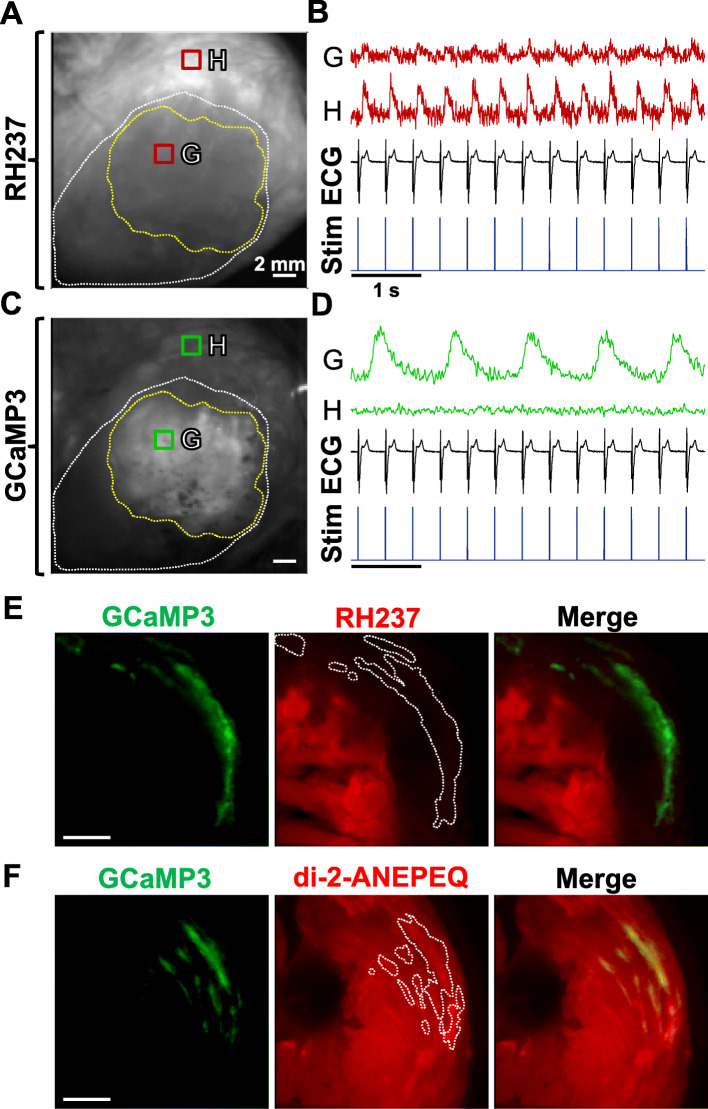
RH237 does not label hESC-CM graft tissue, but di-2-ANEPEQ does label graft. Still images acquired on the “red” RH237 (**a**) and “green” GCaMP3 (**c**) channels from the epicardial surface of a representative cryoinjured heart with GCaMP3+ hESC-CM graft (in this case, derived from the RUES2 line). **b**, **d** Corresponding fluorescence traces on each channel from the two indicated ROIs. The region labeled “G” is located in hESC-CM graft tissue (encircled by the yellow dotted line), while “H” is located in viable host myocardium outside of the cryoinjury zone (encircled by the white dotted line). While RH237-derived oAPs (red traces) from both ROIs occurred in 1:1 synchrony with QRS complexes of the simultaneously-acquired ECG (black) and applied stimulus (blue), GCaMP3 fluorescence transients (green) from graft tissue clearly activated independently from host myocardium. **e** When this heart was transversely sectioned, slices showed uniform staining of the host myocardium by RH237 (red fluorescence), but GCaMP3+ graft tissue (green) was completely devoid of RH237 staining. **f** By contrast, when an equivalently injured and transplanted heart was sectioned following perfusion with di-2-ANEPEQ, comparable dye staining of host and graft tissue was observed

Our group has previously reported that when hESC-CM-engrafted hearts are stained with conventional lipophilic voltage-sensitive fluorescent dyes (e.g., RH237, di-4-ANEPPS), these dyes label host but not graft tissue [13]. To confirm this earlier finding, we transplanted GCaMP3+ hESC-CMs into guinea pig hearts at 10 days post-injury, harvested the engrafted hearts at 2 weeks post-transplantation, and then imaged them ex vivo after perfusion with the dye RH237 (*n* = 3). For these imaging experiments, we used a custom-built, dual-channel, EM-CCD-based system to simultaneously acquire the RH237 (“red” channel) and GCaMP3 (“green” channel) signals. As in our previous work [13], we consistently encountered hearts in which GCaMP3 fluorescence transients activated independently from host myocardium, and yet RH237-derived oAPs from these same graft regions occurred synchronously with the QRS complex of the host ECG (Fig. 1a–d). Hence, while the genetically encoded, graft autonomous indicator of graft activation (GCaMP3) proves that these grafts were in fact uncoupled from the host, the simultaneously acquired voltage dye signal could be erroneously interpreted as indicating 1:1 host-graft coupling. We were able to account for these apparently discordant results by transversely sectioning these hearts after dye labeling and examining them on a dissecting fluorescence stereomicroscope. While all of the examined hearts showed strong, uniform staining of host myocardium by RH237, GCaMP3+ graft regions were entirely devoid of RH237 fluorescence (Fig. 1e). Taken collectively, these observations support our earlier conclusion that graft-derived RH237-derived oAPs from such hearts are factitious, and that these signals instead arise from subendocardial host tissue located beneath the graft that shine through to the epicardial surface [13].

To explain these findings, we hypothesized that lipophilic dyes such as RH237 fail to stain hESC-CM graft tissue because the dye partitions fully into host tissue before reaching the relatively poorly-perfused graft, since hESC-CM grafts are known to have an immature sinusoidal-like vascular supply [41]. If this is correct, we predicted that better labeling of hESC-CM graft tissue might be obtained by the use of a water-soluble voltage-sensitive dye. Water-soluble dyes have been used less commonly in optical mapping experiments because they have to be constantly supplied in the perfusate, but we predicted that this situation might actually be advantageous for the present application because it would allow the dye to penetrate through to the graft. To test this, we transplanted equivalently injured hearts with GCaMP3+ hESC-CMs, harvested engrafted hearts at 2-weeks post-transplantation, then perfused the latter with the water-soluble voltage dye di-2-ANEPEQ (*n* = 3). In contrast to our prior experience with RH237, when these hearts were transversely sectioned and examined, we observed strong, uniform staining of both host and GCaMP3+ graft tissue by di-2-ANEPEQ (Fig. 1f).

### Di-2-ANEPEQ is spectrally separated from the graft-autonomous calcium reporter GCaMP3 and reliably reports myocardial electrical activity

While the preceding observations suggest that di-2-ANEPEQ might be useful as a voltage reporter in hESC-CM graft tissue, additional characterization of the dye was required before proceeding to ex vivo testing of dual-imaging of di-2-ANEPEQ and GCaMP3 fluorescent signals as a strategy to map graft electrical activity. We first used spectrofluorimetry to determine the excitation and emission spectra of di-2-ANEPEQ dye (Supplementary Fig. S2A)**.** Next, we used confocal emission fingerprinting to precisely define the emission spectra of GCaMP3+ and di-2-ANEPEQ-loaded hESC-CMs, both at rest and during depolarization (Supplementary Fig. S2B-D). Taken collectively, these studies showed that both fluorophores can be efficiently excited at 488 nm and that their emission can be readily separated using 500–530 nm band-pass (GCaMP3) and 650 nm long-pass (di-2-ANEPEQ) filters. Further assisting their spectral discrimination, depolarization causes changes in the opposite direction with the preceding filter sets, i.e., cardiomyocyte activation produces an *increase* in GCaMP3 fluorescence intensity but a *decrease* in di-2-ANEPEQ fluorescence (Supplementary Fig. S2E). That said, please note that all di-2-ANEPEQ-derived oAPs hereafter have been inverted (i.e., depicted as -ΔF/F) to match convention.

Because there has been very limited published experience with the use of di-2-ANEPEQ in cardiac optical mapping, we next performed experiments to verify its suitability as a myocardial voltage reporter. For this, we first imaged uninjured (*n* = 5) and injured (*n* = 3) guinea pig hearts without grafts and consistently detected robust di-2-ANEPEQ-derived oAPs. We next correlated host di-2-ANEPEQ-derived oAPs to simultaneously acquired direct electrode recordings [37] and to RH237-derived oAPs. For the latter, RH237 oAPs were acquired by loading and imaging the heart after recording and washout of di-2-ANEPEQ signals. In both cases, we found excellent correlations with di-2-ANEPEQ in terms of activation times, AP morphology, and APDs (*n* = 2) (Supplementary Fig. S3A-F). For example, APD_90_ measurements based on di-2-ANEPEQ and direct electrode recordings were 154.2 ± 2.4 vs 155.5 ± 1.7 ms (*p* = 0.6), while APD_90_ measurements based on RH237 and direct electrode recordings were 146.2 ± 1.5 vs 144.7 ± 1.3 ms (*p* = 0.3). Note that the amplitude of di-2-ANEPEQ fluorescence transients was larger than those obtained with RH237 in the same heart (4.7% vs 2.4% ΔF/F), an encouraging finding given our goal of detecting oAPs in graft tissue.

### Host and graft-derived electrical activity can be reliably detected via the simultaneous imaging of di-2-ANEPEQ (host and graft voltage) and GCaMP3 (graft-only intracellular [Ca^2+^]_i_) fluorescent signals

Having validated the spectral compatibility of di-2-ANEPEQ with GCaMP3 as well as its utility as a myocardial voltage reporter, we next moved to test these two fluorophores in combination for the optical mapping of hESC-CM-engrafted hearts. We hypothesized that di-2-ANEPEQ fluorescence (“red” channel) would report electrical activity in both graft and host tissue, while GCaMP3 fluorescence (“green” channel) would function as a critical graft-autonomous reporter of graft activation. If successful, this approach would provide us a reliable means of distinguishing between host- and graft-derived electrical signals with high spatial and temporal resolution.

To test this approach, we transplanted 1 × 10^8^ GCaMP3+ RUES2 hESC-CMs into cryoinjured guinea pig hearts at 28 days post-injury (*n* = 5). We then harvested engrafted hearts at 2 weeks post-transplantation and imaged them ex vivo using the dual-channel CCD-based system and the experimental protocol depicted in Supplementary Fig. S1. In brief, each heart was perfused ex vivo, mechanically arrested with blebbistatin and then imaged on both channels before perfusion with di-2-ANEPEQ, during perfusion with di-2-ANEPEQ, and during/after dye washout. Hearts were imaged under both spontaneous and paced conditions.

**Fig. 2:**
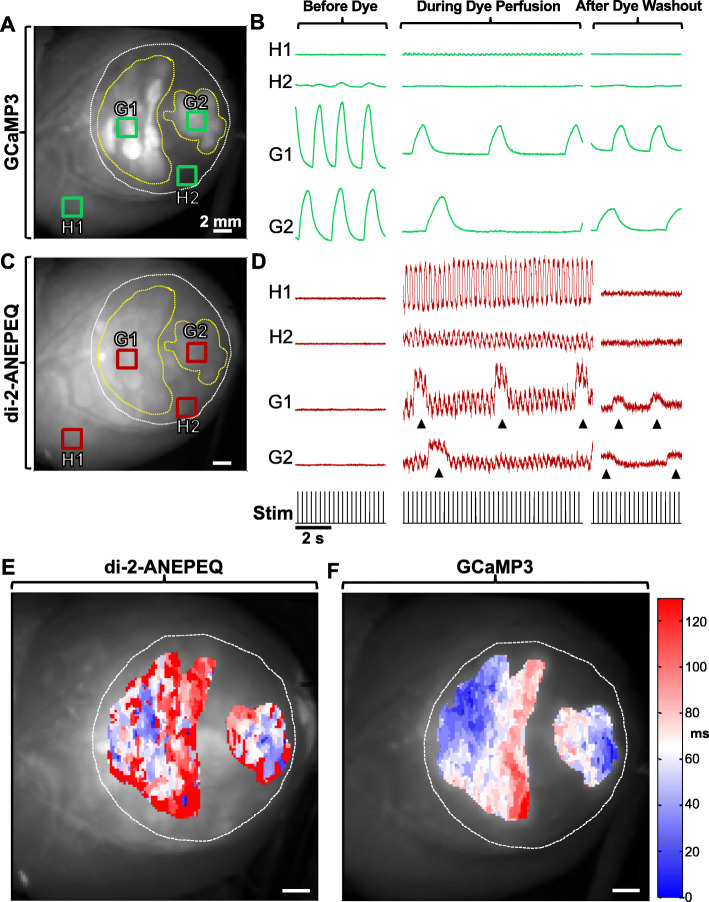
Reliable detection of host and hESC-CM graft electrical activity by simultaneous di-2-ANEPEQ and GCaMP3 imaging. A representative cryoinjured heart with GCaMP3+ hESC-CM graft tissue (RUES2 line) imaged before, during, and after perfusion with di-2-ANEPEQ. **a** Still image depicting the epicardial surface of this heart as acquired on the GCaMP3 (“green”) channel. White and yellow dotted lines indicate the boundaries of the cryoinjury zone and hESC-CM graft tissue respectively, and four ROIs have been selected representing viable host (H1), injured host (H2), and two distinct graft regions (G1 and G2). **b** Mean GCaMP3 fluorescence activity from ROIs identified in **a**, acquired before, during, and after di-2-ANEPEQ perfusion. In this case, both graft regions were uncoupled from the host and from each other, so GCaMP3 fluorescence transients occurred independently of the applied stimuli (black trace below panel **d**). **c** Corresponding still image on the di-2-ANEPEQ (“red”) channel. **d** Simultaneously acquired di-2-ANEPEQ fluorescent signals from these same ROIs before, during, and after di-2-ANEPEQ perfusion. Black arrowheads indicate graft-derived oAPs, seen superimposed over smaller amplitude oAPs from underlying host tissue. **e**, **f** Representative activation maps for graft tissue in this same heart based on di-2-ANEPEQ (**e**) and GCaMP3 (**f**) optical signals. Activation time (in ms) is expressed relative to the first active site within the ROI on a given channel. Note the two graft regions activated independently but have been displayed with the same activation time scale

Figure 2 depicts the findings from a representative imaging experiment obtained from a cryoinjured heart in which the visible hESC-CM graft was uncoupled from the host myocardium (i.e., in which graft-derived GCaMP3 fluorescent transients occurred in no relationship to the host ECG or the applied rate of stimulation). Prior to perfusion with di-2-ANEPEQ dye, we detected strong GCaMP3 fluorescence transients in graft ROIs in the GCaMP3 (“green”) channel with no “bleed-through” of this signal into the di-2-ANEPEQ (“red”) channel (see left-most traces in Fig. 2b, d). Upon perfusion with di-2-ANEPEQ, we observed a differential time-course of dye labeling in host and graft tissue, an outcome consistent with our hypothesis that hESC-CMs grafts have relatively sluggish perfusion. Maximal labeling of host tissue occurred 4.5 ± 0.3 min after the addition of dye to the perfusate, while graft tissue took 13.4 ± 1.1 min to achieve maximal labeling. As expected, ROIs in host myocardium showed di-2-ANEPEQ-derived oAPs that occurred in 1:1 synchrony with the host ECG (and the applied stimuli in the case of paced hearts), and oAPs from host tissue within the injury zone showed a smaller amplitude than oAPs from intact host myocardium (2.4 ± 0.1% vs 4.7 ± 0.3% ΔF/F). At early timepoints following the onset of dye perfusion, graft ROIs also showed equivalently timed but smaller-amplitude oAPs that we attribute to host tissue located deep under the graft. However, over time, these same graft ROIs showed the gradual emergence of *graft-derived* oAPs that were superimposed on the continuing host-derived oAPs (see the middle traces in Fig. 2b, d). Proving their graft origin, these graft-derived oAPs always occurred synchronously with the simultaneously acquired, graft-autonomous GCaMP3 fluorescence transients and not necessarily with the host ECG, as expected for intra-cardiac grafts that may or may not be coupled with host myocardium [13, 14]. The durations of graft-derived oAPs were also distinct from their counterparts in host tissue, as detailed further below.

Interestingly, we observed the reverse sequence with regard to dye washout from host and graft tissues. Host tissues lost all detectable oAPs within 13.6 ± 1.6 min after the switch to di-2-ANEPEQ-free perfusate, while graft tissue showed graft-derived oAPs—now no longer with the superimposed host-derived oAPs—that persisted for at least 30 min. At this late time point, graft-derived oAPs were typically smaller in amplitude relative to those before washout, but they still had a mean ΔF/F of 2.3 ± 0.2% that allowed for reliable quantitation (see right-most traces in Fig. 2b, d for representative traces during dye washout). This differential tissue labeling over time provided us with two independent means to reliably discriminate between graft- and host-derived signals. First, we correlated between graft-derived oAPs based on di-2-ANEPEQ and the simultaneously acquired graft-autonomous GCaMP3 signals and always found good agreement between the two reporters. Graft cycle lengths measured independently by the two reporters showed a correlation coefficient of *R*^2^ = 0.995, and, as shown in Fig. 2e, f, graft activation maps recorded on the “red” and “green” channels showed similar patterns of spatial activation. Second, by performing serial dye wash-in and washout experiments, we were able to reliably identify graft- and host-derived di-2-ANEPEQ signals.

To further validate the reliability of this system to distinguish between host- and graft-derived electrical signals, we attempted direct microelectrode recordings of engrafted hearts during dual-imaging of GCaMP3 and di-2-ANEPEQ. While technically very challenging, we were able to simultaneously acquire direct electrode recordings from graft tissue in a single heart (of 4 attempted). In this case, we observed excellent temporal agreement between the di-2-ANEPEQ and directly recorded APs, with graft APD_90_ based on di-2-ANEPEQ data being 232.6 ± 4.8 ms, and APD_90_ derived from direct electrode recordings being 234.0 ± 3.0 ms (Supplementary Fig. S4).

### Optical mapping outcomes from injured hearts transplanted with GCaMP3+ hESC-CM grafts

Given this capacity to reliably detect and distinguish between host- and graft-derived electrical activity, we advanced to experiments to investigate how parameters including cell line, the timing of cell transplantation, and spontaneous versus paced conditions might affect the electrical behavior of engrafted hearts. To examine this, we transplanted GCaMP3+ hESC-CMs from each of the three cell lines (RUES2, H7, and ESI-17) into cryoinjured guinea pig hearts at either 10 or 28 days post-injury (*n* = 3–5 per condition). Due to restrictions that arose during experiments, the RUES2 line was not tested at the 10-day timepoint. Qualitatively similar di-2-ANEPEQ dye wash-in and wash-out kinetics was noted across all conditions, and grafts from all lines showed similar oAP amplitudes.

**Fig. 3:**
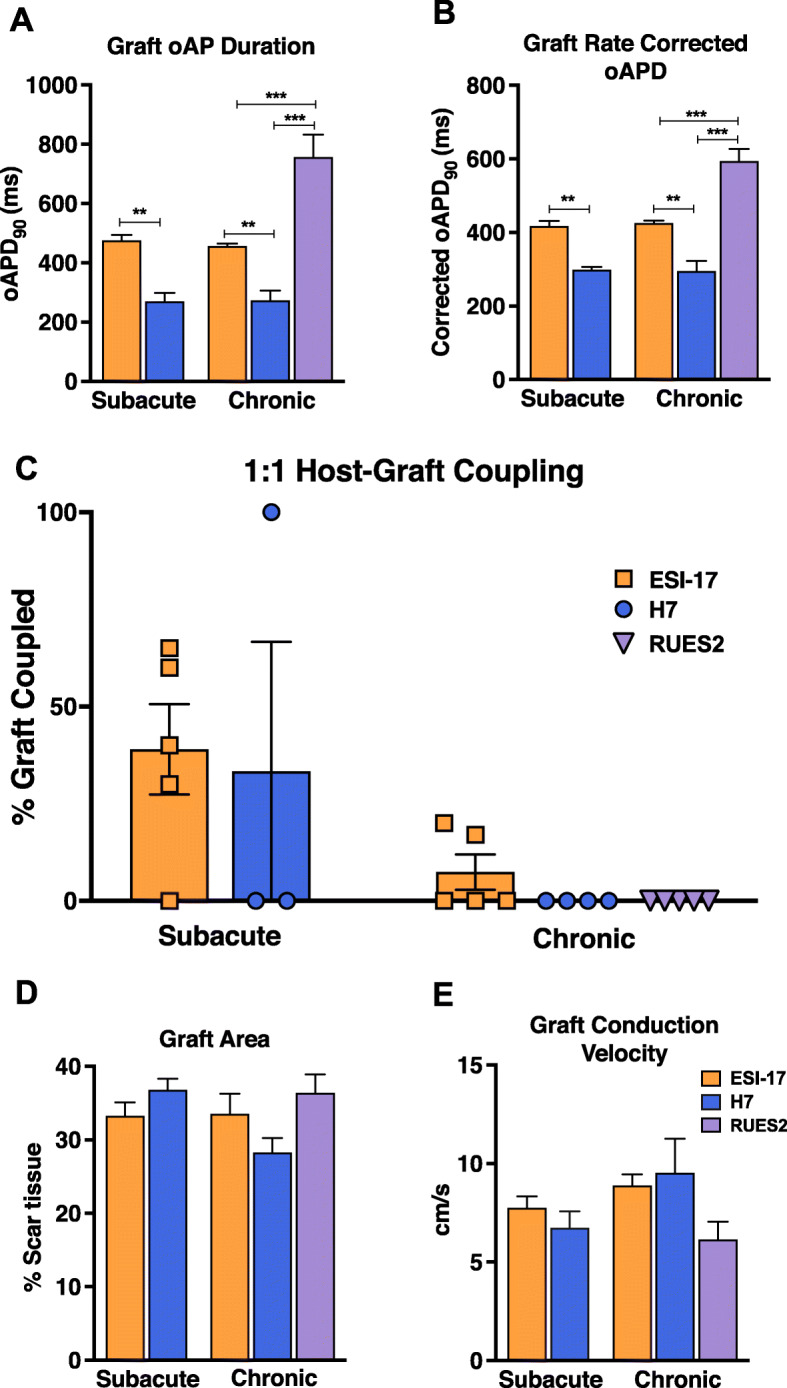
Electrophysiological properties of hESC-CM graft tissue from three GCaMP3+ lines after transplantation into injured hearts. Optical mapping outcomes by experimental model (subacute versus chronic) for hESC-CMs derived from three different parental hESC lines. **a** Mean graft optical action potential durations at 90% repolarization (oAPD_90_). **b** Mean graft oAPD_90_ for these same experimental conditions after applying Fridericia’s rate correction. **c** Percentage of hESC-CM graft area visible from the epicardial surface in each recipient heart that showed 1:1 host-graft coupling during imaging at 2 weeks post-transplantation. **d** Mean graft area, expressed as the percentage of the injury area occupied by graft as viewed from the epicardial surface. **e** Mean graft conduction velocity. *n* = 3–5 hearts per condition, **p < 0.01, ***p < 0.001

That said, graft formed using the different parental lines showed striking differences in other key electrophysiological parameters. First, all RUES2 and ESI-17 hESC-CM grafts displayed very long oAPDs (APD_90_ of 756 ± 76 ms and 464 ± 8 ms, respectively) that were not observed in grafts formed with their H7 counterparts (APD_90_ of 272 ± 21 ms, *p* < 0.01) (Fig. 3a). These differences in oAPD persisted after correcting for differences in firing rate (with mean rate-corrected APD_90_ values of 594 ± 33 ms, 429 ± 7 ms vs 297 ± 15 ms for RUES2, H7, and ESI-17 hESC-CM graft, respectively (Fig. 3b). There were also major differences in the electromechanical integration of grafts formed with each of the three parental hESC lines (Fig. 3c). Across the 22 hearts imaged across all conditions, 7 hearts (or 32%) had at least one graft region that was found to be coupled with host myocardium. Consistent with our prior reports [13, 14], host-graft coupling was typically more extensive in the case of grafts formed in the subacute (10 days post-injury) versus chronic (28 days post-injury) model. In the subacute injury model, we found at least some regions of coupled graft in 4 of 5 ESI-17 hESC-CM recipients and 1 of 3 H7 hESC-CM recipients. When analyzed on a per heart basis, a mean of 34.8% of the total visible graft area was found to be 1:1 coupled in ESI-17 recipients versus 33.3% in H7 recipients. Coupling was consistently poor in the chronic injury model across all three lines. Here, only 2 of 5 ESI-17 hESC-CM recipients showing any regions of 1:1 coupled graft (corresponding to a mean of 16.6% of the visible graft area per heart), while no regions of 1:1 coupled graft were detected whatsoever in either H7 or RUES2 hESC-CM recipients.

**Fig. 4:**
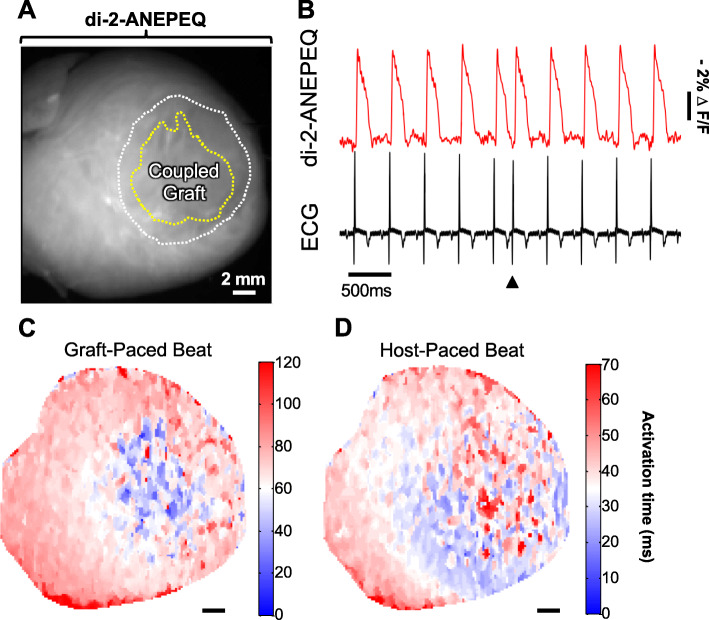
Optical mapping with di-2-ANEPEQ confirms hESC-CM grafts can act as an ectopic pacemaker. A cryoinjured heart with GCaMP3+ H7 hESC-CM graft was imaged under spontaneous conditions (no external pacing), and the graft was found to pace host myocardium. **a** Still image showing the epicardial surface of the heart on the di-2-ANEPEQ channel with the boundaries of cryoinjury scar and graft indicated by white and yellow dotted lines, respectively. Base of the heart to the upper left, apex to lower right. **b** di-2-ANEPEQ fluorescent signals and ECG trace for this heart with the timing of the ectopic graft-paced beat depicted in panel **c** indicated by the black arrowhead. **c** Activation map for a representative graft-paced beat, showing the earliest activating area (blue) starts in the coupled graft footprint, then propagates outward towards the LV base and apex (white to red). **d** Activation map for a representative host-paced beat, with earliest activation (blue) initiating at the LV apex, then propagating towards the LV base and finally back apically to activate the graft (white to red). Note the slower kinetics of the graft-paced versus host-paced beat. Also, note that both activation maps represent information from a *single* beat


Although grafts formed using hESC-CMs from the three different parental lines showed differences in their oAPDs and host-graft coupling outcomes, we did not find significant differences in other parameters including visible graft area (Fig. 3d) or graft CV (Fig. 3e). Interestingly, graft CV in all hearts was significantly slower than in adjacent host myocardium (< 25%). In addition to slow graft CVs that could potentially drive reentrant arrhythmias, all imaged grafts showed instances of other potentially pro-arrhythmic behavior including propagation along vectors opposite from that in host tissue and/or spatial patterns of activation that varied from beat-to-beat (see Supplementary Movie File 1). Moreover, in one heart with GCaMP3+ H7 hESC-CM graft, we found that the graft was 1:1 coupled with the host, but that it was the graft that paced the host rather than the other way around. Indeed, when imaged a time-point at which both host and graft tissue were loaded with di-2-ANEPEQ, electrical activation in graft was actually found to precede that of host myocardium (Supplementary Movie File 2, Fig. 4a–d).

**Additional file 7: Supplementary Movie File 2.** GCaMP3+ hESC-CM graft acting as an ectopic pacemaker. A cryoinjured heart with GCaMP3+ H7 hESC-CM graft was harvested at 14 days post-transplantation, loaded with di-2-ANEPEQ, and imaged ex vivo during spontaneous beating (i.e., no external pacing). This video shows the dynamic epicardial di-2-ANEPEQ fluorescent signal, which reports electrical activation in both host and graft tissue, as well as the time-synchronized host ECG, both displayed at one-twentieth actual speed. The heart is oriented with the apex on the right and the base on the left. Host and graft voltage activation have been pseudo-colored with black indicating rest and white depolarization. Note that during the first, second, and fourth beats shown, electrical activation actually begins first in hESC-CM graft tissue located near the center of the heart. Only during the third beat, in what could otherwise be interpreted as a premature ventricular contraction, does electrical activation initiate normally in host tissue near the left ventricular apex.

### Structure and gap junction expression in hESC-CM-engrafted hearts by histology

**Fig. 5:**
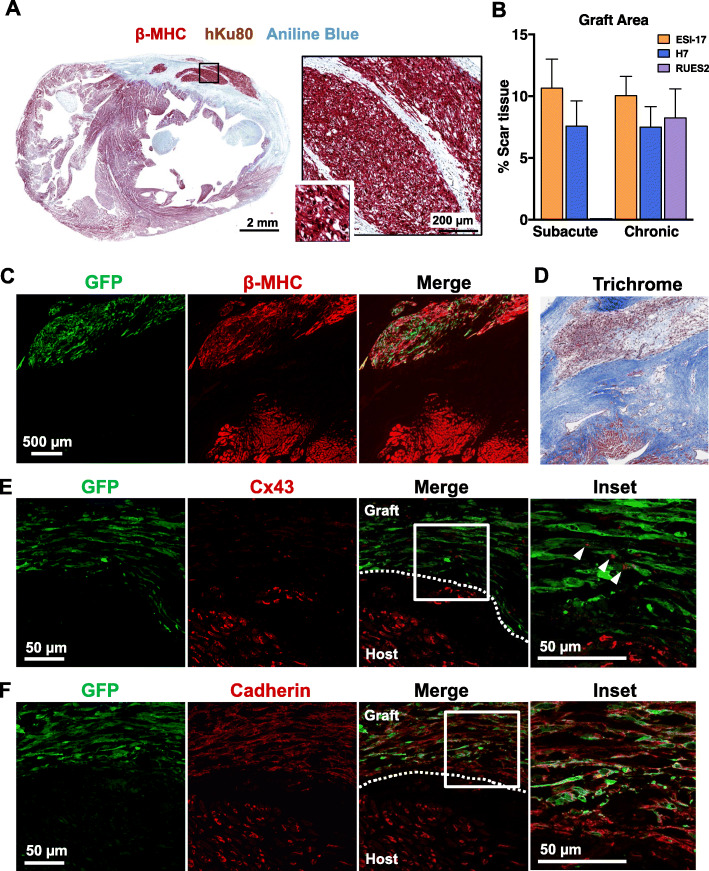
Histological analysis of injured hearts with GCaMP3+ hESC-CM grafts. **a** Representative cryoinjured heart with GCaMP3+ ESI-17 hESC-CM graft immunostained for beta myosin heavy chain (β-MHC) (muscle, red) and human-specific Ku80 nuclear protein (graft nuclei, brown) and counterstained with aniline blue (scar tissue, blue). Graft tissue within the box is shown at higher magnification to the right. **b** Graft size by histomorphometry, expressed as the percentage of injury area occupied by graft. *n* = 3–5 hearts per condition. **c** Confocal photomicrograph of cryoinjured heart with GCaMP3+ H7 hESC-CM graft near the border zone, immmunostained for GCaMP3+ (anti-GFP antibody, green) and β-myosin heavy chain (β-MHC, red). **d** Adjacent histological section stained with Masson’s trichrome stain. **e** Confocal photomicrograph of GCaMP3+ H7 hESC-CM graft near border zone immunostained for GCaMP3+ (anti-GFP antibody, green) and Cx43 (red). While grafts consistently exhibited a lower level of Cx43 expression than host myocardium, occasional scattered Cx43 gap junction plaques were observed (white arrowheads). **f** Adjacent histological section immunostained for GCaMP3+ and N-cadherin (red). Qualitatively similar patterns of Cx43 and N-cadherin expression were observed by grafts formed by ESI-17 and RUES2 hESC-CMs, as well as grafts with or without GCaMP expression (data not shown)

Our group has previously examined in detail the composition of hESC-CM grafts as well as their structural effects on recipient hearts [9, 12–14, 16, 42], but here we performed focused histological studies intended to specifically address whether differences in scar size, graft size, graft distribution, or intercalated disc structure might account for the observed differences in the electrical behavior of grafts formed with cardiomyocytes derived from different hESC parental lines. As in our previous work, all hESC-CM recipient hearts showed irregular grafts of human myocardium that were mostly located within the cryoinjury scar (Fig. 5a–d), and we observed no significant obvious differences in terms of scar size or graft area between experimental groups. Interestingly, there was no obvious correlation between the presence or extent of host-graft coupling by optical mapping and either graft size or distribution by histology. Indeed, we found multiple hearts in which there was abundant host-graft contact by histology and no 1:1 host-graft coupling during mapping, and conversely, other instances in which the majority of the graft was isolated in scar tissue by histology and yet was coupled.

To rule out a difference in intercalated disc structure between grafts formed with cardiomyocytes from different hESC-CM lines, we immunostained recipient hearts with antibodies against the major gap junction protein connexin-43 (Cx43) and the adherens junction protein N-cadherin. Consistent with our prior work [9, 13, 17], we found Cx43 in graft tissue formed from all three lines to be lower than in adjacent host myocardium and, where present, to be uniformly distributed throughout the sarcolemma rather than localized to the intercalated discs (Fig. 5e). Grafts formed from all three lines also showed comparable levels of N-cadherin expression, in this case, with strong immunoreactivity that approached that of host myocardium but again lacked subcellular localization to the intercalated discs (Fig. 5f).

### GCaMP3 expression can prolong the action potential duration of hESC-CMs

**Fig. 6:**
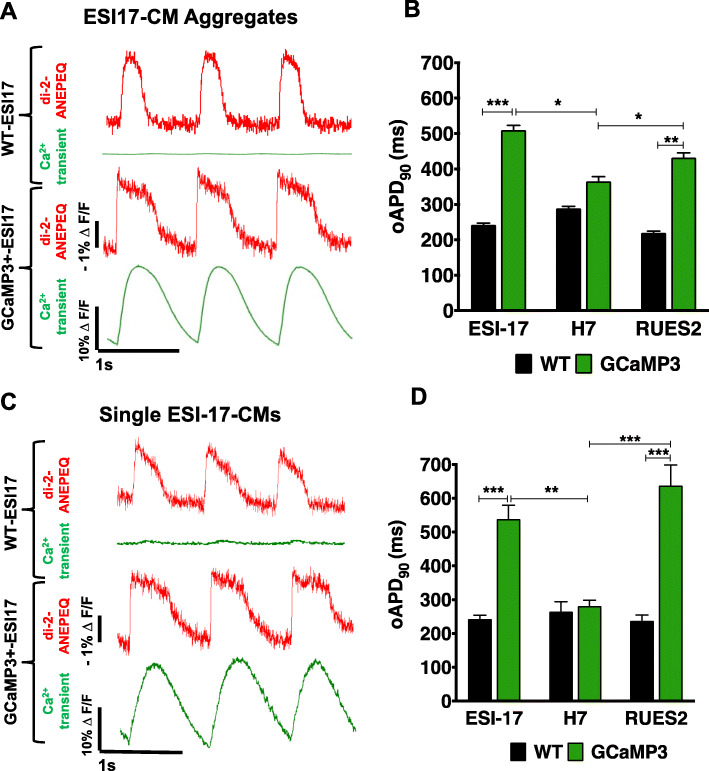
Optical action potentials in WT versus GCaMP3+ hESC-CMs in vitro. **a** Fluorescence traces derived from representative WT (upper) or GCaMP3+ (lower) ESI-17 hESC-CM aggregates simultaneously imaged on the di-2-ANEPEQ (“red,” voltage) and GCaMP3 (“green,” intracellular calcium) channels during pacing at 1 Hz. **b** Mean oAPD_90_ values for WT versus GCaMP3+ hESC-CM aggregates generated from each of the three parental lines. **c**, **d** Corresponding fluorescence traces from representative individual WT (upper) or GCaMP3+ (lower) ESI-17 hESC-CMs, as well as mean oAPD_90_ values for WT versus GCaMP3+ hESC-CMs from each of the three parental lines when recorded as single cells. Note that, using both preparations, GCaMP3+ cardiomyocytes from the ESI-17 and RUES2 hESC lines showed significantly longer oAPDs than their WT counterparts. Results are from *n* > 12 recordings per condition using cells from at least three differentiation runs. *p < 0.05, **p < 0.01, ***p < 0.001

In prior work, our group has used patch-clamp techniques to obtain measurements of APD in WT hESC-CMs on the order of ~ 200 ms [8, 40]. Our finding here of unusually long APDs in both GCaMP3+ RUES2 and ESI-17 hESC-CM grafts led us to speculate that GCaMP3 expression itself might be contributing “off-target,” AP-prolonging effects that might also be observed in cardiomyocytes prior to transplantation. To test this hypothesis, we performed in vitro optical voltage recordings from individual or submillimeter aggregates of WT versus GCaMP3+ hESC-CMs generated from each of the three parental hESC lines. For this, cells and aggregates were stained with the fluorescent voltage-sensitive dye di-2-ANEPEQ, then their oAPs were imaged during field-stimulation. Consistent with our earlier in vivo observations, WT and GCaMP3+ H7 hESC-CMs showed similar oAPDs, but GCaMP3-expressing cardiomyocytes from the ESI-17 and RUES2 hESC lines both showed significantly prolonged oAPDs relative to their respective WT counterparts (Fig. 6 and Supplementary Fig. S5A&B). This AP-prolonging effect was observed in GCaMP3+ hESC-CMs derived from multiple transgenic clones from the affected lines (Supplementary Fig. S5C) and with multiple methods of cardiac differentiation (data not shown).

## Discussion

hESC-CMs are a promising cell source for potential novel regenerative therapies, but concerns about the risk of graft-related arrhythmias have highlighted the need for new tools to investigate their electrophysiological properties in vivo. Optical mapping is an attractive experimental modality for this application, and our efforts to apply this technology here led to several observations of interest. First, we confirmed our earlier findings that it can be very challenging to distinguish between hESC-CM graft- and host-derived optical voltage signals; in fact, in our hands, conventional lipophilic voltage dyes do not label hESC-CM graft tissue at all. To overcome this problem, we tested a novel strategy based on the simultaneous imaging of a graft-autonomous reporter of activation (hESC-CM grafts expressing GCaMP3) and the water-soluble voltage dye di-2-ANEPEQ (which stains both graft and host). In brief, after a sufficient duration of perfusion with di-2-ANEPEQ, graft regions showed the superposition of two signals: graft-derived oAPs (that always occurred in synchrony with the graft-autonomous GCCaMP3 signal) and host-derived oAPs (that occurred in synchrony with applied stimulation and resulted from host myocardium located deep to the graft that shined through the scar to the epicardial surface). However, during washout, labeling was much more rapidly lost from host myocardium, resulting in the transient isolation of graft-derived oAPs (although the amplitude of these graft-derived oAPs is typically diminished from the peak of dye labeling). Hence, while problematic for the longer-term goal of remuscularizing the injured heart with electrically integrated new myocardium, the phenomenon of uncoupled hESC-CM grafts (such as that depicted in Fig. 2) was actually useful for the purposes of demonstrating the reliability of our approach because both the GCaMP3 and graft-derived di-2-ANEPEQ signals were synchronized with each other throughout but they occurred in no relation to electrical activation in host muscle. Interestingly, however, uncoupled grafts do appear to fluctuate somewhat in the periodicity of their spontaneous firing over time, again as illustrated by the time-course experiment depicted in Fig. 2.

This approach allowed us to acquire the first, unambiguously *graft-derived* oAPs from hESC-CM tissue in injured hearts, and to reveal potentially pro-arrhythmic behavior by hESC-CM graft tissue including incomplete host-graft coupling, slow graft CVs, spatially abnormal patterns of graft activation that varied from beat to beat, and graft-induced ectopy. That said, our imaging system has a number of important advantages and disadvantages that warrant further discussion. The principal advantage of our approach is that it provides two independent means of reliably distinguishing between graft- and host-derived electrical signals: both the simultaneously-acquired, graft-autonomous GCaMP3 signal and the option of performing serial wash-in and wash-out experiments to isolate graft-derived oAPs via the differential labeling kinetics of di-2-ANEPEQ in host and graft tissue. The differential time-course of di-2-ANEPEQ labeling between graft and host is likely a consequence of the relatively poor perfusion of graft tissue, and this fact should be kept in mind during any experiments in which engrafted hearts are treated with pharmacological agents that may take longer to reach the graft. Another potential advantage of di-2-ANEPEQ is that, as a water-soluble dye, it can be used without solvents such as DMSO which are required to deliver conventional lipophilic dyes.

Our approach of dual-imaging di-2-ANEPEQ and GCaMP3 signals also has its limitations. First, in contrast to lipophilic dyes that are typically introduced as a bolus [43], di-2-ANEPEQ must be constantly perfused through the heart (via a recirculation loop) to acquire oAPs. While we did not observe differences in the electrical activity of hearts loaded with either di-2-ANEPEQ or RH237, off-target effects of dyes and other agents are always a potential concern when continuously applied for long periods of time. Second, wash-in and wash-out experiments can be technically challenging to perform, and they provide a relatively narrow time-window for pharmacological or electrophysiological interventions because graft oAPs can only be reliably isolated and detected for ~ 10 min during dye wash-out. Further, serial wash-out experiments increase the total duration of each imaging study, necessitating an ex vivo heart preparation that is stable for at least a couple of hours. The GCaMP3 fluorescent signal is also subject to some degree of photobleaching over longer experimental timelines.

It should be noted that we used blebbistatin to mechanically arrest hearts during imaging, and perfusion with this agent causes a high-degree of autofluorescence in myocardium at wavelengths that overlap with GCaMP3 [13, 44, 45]. In our own experiments, we found that this actually helped to accentuate the border between viable muscle and the cryoinjury scar since the background fluorescence caused by blebbistatin was significant only in intact myocardium. However, if graft were ever large enough to generate a macroscopic motion (not the case here), poor penetration of the uncoupler into the scar and graft might result in motion artifacts that could affect the shape of the graft oAP. Next, while blebbistatin has been extensively used in past electrophysiological studies with pluripotent stem cell-derived cardiomyocytes in vitro without reports of significant off-target effects [46–51], we cannot rule out the possibility of such effects in vivo or that subtle line-specific differences that may have contributed to the graft outcomes reported here.

Arguably the most important limitation of the approach developed in this study (but also an important note of caution for the field) is that GCaMP3 expression itself appears capable of significantly altering the electrophysiological properties of hESC-CMs. Indeed, in our hands, GCaMP3 exerted significant AP-prolonging effects on cardiomyocytes derived from two different hESC lines (RUES2 and ESI-17), both in vitro and after transplantation into injured hearts. AP prolongation was observed in recordings from both individual transgenic cardiomyocytes and submillimeter multicellular aggregates. This phenomenon was unexpected, as to our knowledge, no one has previously reported such off-target effects by GCaMP3 expression. Indeed, transgenic mice created with systemic and cardiomyocyte-restricted overexpression of the closely related GCaMP molecule GCaMP2 had seemingly normal cardiac function [19]. While beyond the scope of the present study, we intend future work to investigate the mechanistic basis of this AP prolongation in GCaMP3+ RUES2 and ESI-17 hESC-CMs, including the examination of individual ionic currents in WT versus transgenic myocytes under voltage-clamp.

Notwithstanding these limitations, the imaging approach developed in the present study allowed us to obtain important new insights into the electrophysiological properties of hESC-CM grafts in injured hearts. In addition to acquiring previously unknown parameters such as graft CV and APD, we were able to directly observe complex host-graft electrical interactions and extrapolate to outcomes at the organ level. For example, in the case of the heart depicted in Supplementary Movie File 2 and Fig. 4, we found that the same graft could at various time-points either reliably capture and follow the host rhythm or act as an ectopic pacemaker driving the host tissue instead. To our knowledge, this represents the first *direct* demonstration of graft ectopy (e.g., by optical methods), but it is entirely consistent with recent observations from our group based on electroanatomic mapping following hESC-CM transplantation in a swine infarct model [17]. The fact that this ectopy was episodic also has important implications to the arrhythmogenic potential of hESC-CMs, and it would be informative to conduct similar experiments during challenge with physiological or pharmacological stressors. This behavior also underscores the value of optical mapping with separate graft and host reporters, as it can otherwise be quite challenging to discern exactly what is driving what, especially under conditions in which both graft and host have similar intrinsic rates [13].

Another finding of interest was the relative low level of host-graft integration, particularly with hESC-CM grafts derived from two of the three hESC lines tested (H7 and RUES2). There are multiple factors that could have contributed to this outcome. One obvious explanation for the lack of host-graft coupling would be an absence of physical contact between host and graft, but this parameter did not obviously vary in histological sections between grafts from different lines. We also speculate that failure to couple could result from a *functional* block to integration. Such a block might be due to low levels of Cx43 gap junction expression in graft tissue and/or delays in recovery and depolarization after a preceding long AP (as was frequently observed in these experiments). Low expression and/or isotropic distribution of Cx43 gap junctions may have also contributed to the very slow graft CVs found in this study (with a mean graft CV of < 10 cm/s for each of the three hESC lines versus ~ 50 cm/s for guinea pig LV myocardium [52].)

Finally, we anticipate that the optical approaches developed in this report will be very useful for testing new approaches to improve graft electrical integration and function (e.g., transplantation of more mature hESC-CMs or hESC-CMs with enhanced Cx43 gap junction expression), as well as for exploring how recipient parameters may affect these same outcomes. For example, while here we used male and relatively young (~ 8-week-old) animals in the present experiments, we intend future work to address how graft electrophysiological function may differ in male versus female or young versus aged recipients. Next, while we have compared outcomes in both subacute and chronic cardiac injury models, it will be important to investigate how co-morbidities or pharmacological treatments that might be encountered in human patients with ischemic cardiomyopathy (e.g., chronic ischemia, inotropic drugs) may affect graft electrophysiology. Finally, we are particularly interested to understand the molecular basis of the line-to-line differences in electrophysiological behavior noted in vivo (for example, the shorter oAPDs and apparently better host-graft electromechanical integration of H7 hESC-CM grafts as depicted in Fig. 3). Numerous investigators have previously described significant line-to-line variation in the electrophysiological phenotype of pluripotent stem cell-derived cardiomyocytes in vitro [53, 54], perhaps as a consequence of epigenetic “memory” [55], and we observed similar heterogeneity when recording oAPs from hESC-CM or hESC-CM aggregates in vitro (Fig. 6). It is thus perhaps unsurprising that such variation would be comparable (or even magnified) in vivo. At present, we can only speculate that this variation reflects underlying differences in ion channel and/or gap junction expression in graft cardiomyocytes, but we anticipate future studies that will correlate optical mapping outcomes with transcriptomics on graft myocardium.

## Conclusions

In conclusion, this study described a novel optical strategy to investigate the electrical behavior of injured hearts with hESC-CM graft tissue based on the simultaneous imaging of GCaMP3 and the water-soluble voltage dye di-2-ANEPEQ. Using this approach, we observed multiple potentially pro-arrhythmic behavior by hESC-CM grafts including incomplete host-graft electromechanical integration, graft ectopy, and slow graft CVs that could predispose to reentry. Grafts tissue formed with cardiomyocytes from different lines showed significant heterogeneity in electrophysiological parameters; for example, RUES2 and ESI-17 hESC-CMs both showed long APDs that appear to be attributable at least in part to GCaMP3 expression. We expect that application of the tools and models described in this report will provide future insights into the electrophysiology of intra-cardiac grafts and will prove useful in testing strategies to further improve graft integration and electrical function.

## Supplementary information

**Additional file 1: Supplementary Fig. S1.** Experimental imaging protocol and cardiac optical mapping system. **A**: Overview of typical experimental protocol used to image hearts ex vivo before, during and after perfusion with di-2-ANEPEQ. **B&C:** Schematic of the dual EM-CCD-based imaging system (**B**) with details of the filter set used for excitation (**C**). In brief, excitation light was collimated and bandpass filtered to 450–490 nm before being reflected onto the Langendorff-mounted heart. Emitted light was collected and split into the “green” and “red” channels first by a 565 nm longpass dichroic mirror inside the DC2 dual-channel splitter. The “green” channel (i.e., GCaMP3) signal was then further bandpass filtered to 500–530 nm before being imaged by an EM-CCD camera, while the “red” channel (i.e., voltage dye) signal was longpass filtered at 650+ nm for di-2-ANEPEQ imaging or 716+ nm for RH237 imaging before being imaged on a separate EM-CCD camera operated simultaneously.

**Additional file 2: Supplementary Fig. S2.** Spectra of GCaMP3 and di-2-ANEPEQ in hESC-CMs in vitro**. A:** Absorbance (ABS) and emission (EM) spectra of di-2-ANEPEQ by spectrofluorimetry after loading into hESC-CMs. Note di-2-ANEPEQ-loaded hESC-CMs absorb with a peak at ~ 488 nm and emit with a peak at ~ 600 nm. **B:** Emission spectra of WT hESC-CMs before and after loading with di-2-ANEPEQ, as determined by confocal lambda scanning. **C:** Emission spectra of GCaMP3+ hESC-CMs during systole and diastole (solid and dotted traces, respectively). **D:** Emission of GCaMP3+ hESC-CMs after loading with di-2-ANEPEQ as recorded in both systole and diastole. GCaMP3 mediates a large increase in emission signal during systole, while di-2-ANEPEQ emission exhibits a small spectral shift to the left (best seen in the magnified inset depicting signal at longer wavelengths). The shaded areas denote filter sets selected to separate these fluorophores in all subsequent experiments. **E:** Mean fluorescence activity over time in a single GCaMP3+ hESC-CM after loading with di-2-ANEPEQ, using the filter sets depicted in panel **D**. Note that while GCaMP3 fluorescence increases upon depolarization, di-2-ANEPEQ fluorescence decreases at the emission wavelengths selected for detection (> 650 nm).

**Additional file 3: Supplementary Fig. S3**. di-2-ANEPEQ reliably reports myocardial electrical activity. Still images and fluorescence traces on the “red” channel from a naïve heart (uninjured and not transplanted) acquired after di-2-ANEPEQ loading (**A&B**), after di-2-ANEPEQ washout (**C&D**), and then after loading with RH237 (**E&F**). These fluorescent oAPs (red traces) were recorded simultaneously and showed good temporal agreement with direct voltage recordings via sharp electrode (black traces). **G.** Still image (left) and voltage activation map (right) from a naïve heart.

**Additional file 4: Supplementary Fig. S4.** Simultaneous di-2-ANEPEQ imaging and direct electrode recording of hESC-CM graft electrical activity. A cryoinjured heart with hESC-CM graft (H7 line) was impaled with a sharp electrode within the graft tissue to correlate the di-2-ANEPEQ-derived voltage signal with direct intracellular voltage recordings. **A:** Epicardial still image taken on the di-2-ANEPEQ channel, showing the uncoupled hESC-CM graft footprint (yellow dotted line) inside the cryoinjury region (white dotted line) and the recording electrode positioned in graft tissue. Two ROIs are indicated: one in host myocardium outside the cryoinjury zone and one in hESC-CM graft tissue overlying the tip of the recording electrode. **B:** Simultaneously acquired oAPs for these two ROIs, as well as the simultaneously acquired direct voltage and ECG recordings. Note the excellent temporal correlation between the direct electrode recordings and di-2-ANEPEQ-derived oAPs from this hESC-CM graft, which was uncoupled from the host.

**Additional file 5: Supplementary Fig. S5**. In vitro optical action potentials from cardiomyocytes from multiple hESC lines. **A, B:** Representative fluorescence traces from WT or GCaMP3 cardiomyocyte aggregates generated from either the H7 (**A**) or RUES2 (**B**) hESC lines. Aggregates were simultaneously imaged on the di-2-ANEPEQ (“red”, voltage) and GCaMP3 (“green”, intracellular calcium) channels during pacing at 1 Hz. While oAPD was similar between WT and GCaMP3+ H7 hESC-CM aggregates, GCaMP3+ aggregates from the RUES2 line typically showed oAPD prolongation relative to their WT counterparts. **C:** To rule out the possibility that the apparent AP-prolonging effect of GCaMP3 was just reflective of idiosyncratic hESC clones, oAPs were acquired from hESC-CM aggregates formed from four different GCaMP3+ ESI-17 hESC lines. Cardiomyocytes from all four clones showed similar increases in oAPD relative to WT controls.

**Additional file 6: Supplementary Movie File 1.** GCaMP3+ hESC-CM grafts commonly activate along vectors distinct from host tissue and with variable spatial propagation patterns. Representative cryoinjured and GCaMP3+ hESC-CM engrafted heart with the graft exhibiting beat-to-beat changes in spatial activation that are all distinct from the direction of activation in host tissue (host tissue activation not shown). In this movie, graft activation from the GCaMP3 channel is shown at one tenth speed over a still image of the injured heart oriented with the apex at the right, base at the left. Graft activation is pseudocolored black to indicate resting tissue, and white to indicate depolarized tissue. Note this GCaMP3+ H7 hESC-CM graft was uncoupled from the host heart.

## Data Availability

The datasets used and/or analyzed during the current study are available from the corresponding author upon reasonable request.

## Abbreviations

AP: Action potential
APD: Action potential duration
APD_90_: Action potential duration at 90% repolarization
CV: Conduction velocity
ECG: Electrocardiogram
FOV: Field of view
fps: Frames per second
hESC-CM: Human embryonic stem cell-derived cardiomyocyte
LV: Left ventricular
oAP: Optical action potential
oAPD: Optical action potential duration
ROI: Region of interest
WT: Wild-type
