# Correction to: Optical mapping of human embryonic stem cell-derived cardiomyocyte graft electrical activity in injured hearts

**DOI:** 10.1186/s13287-020-02077-9

**Published:** 2021-01-19

**Authors:** Dominic Filice, Wahiba Dhahri, Joell L. Solan, Paul D. Lampe, Erin Steele, Nikita Milani, Benjamin Van Biber, Wei-Zhong Zhu, Tamilla Sadikov Valdman, Rocco Romagnuolo, José David Otero-Cruz, Kip D. Hauch, Matthew W. Kay, Narine Sarvazyan, Michael A. Laflamme

**Affiliations:** 1 Department of Bioengineering, University of Washington, Seattle, WA 98195 USA; 2 Institute for Stem Cell & Regenerative Medicine, University of Washington, Seattle, WA 98195 USA; 3 McEwen Stem Cell Institute, University Health Network, 101 College Street, Rm 3-908, Toronto, ON M5G 1L7 Canada; 4 Peter Munk Cardiac Centre, University Health Network, Toronto, ON M5G 2N2 Canada; 5 Fred Hutchinson Cancer Research Center, Seattle, WA 98109 USA; 6 Department of Biology, University of Washington, Seattle, WA 98195 USA; 7 Department of Pathology, University of Washington, Seattle, WA 98195 USA; 8 Department of Biomedical Engineering, G. Washington University, Washington, DC 20052 USA; 9 Department of Pharmacology & Physiology, G. Washington University, Washington, DC 20052 USA; 10 McEwen Stem Cell Institute, University Health Network, 101 College Street, Rm 3-908, Toronto, ON M5G 1L7 Canada; 11 Peter Munk Cardiac Centre, University Health Network, Toronto, ON M5G 2N2 Canada; 12 Department of Laboratory Medicine & Pathobiology, University of Toronto, Toronto, ON M5G 1L7 Canada

**Correction to: Stem Cell Res Ther 11, 417 (2020)**

**https://doi.org/10.1186/s13287-020-01919-w**

The original article [1] contained a typo in co-author, Rocco Romagnuolo’s name which has since been corrected.

## References

[1] Filice D, Dhahri W, Solan JL (2020). Optical mapping of human embryonic stem cell-derived cardiomyocyte graft electrical activity in injured hearts. Stem Cell Res Ther.

