# Supplementary figures and images for: Optical mapping of human embryonic stem cell-derived cardiomyocyte graft electrical activity in injured hearts
Source: Stem Cell Res Ther. 2020 Sep 25;11:417. doi: 10.1186/s13287-020-01919-w (PMC7523067; doi:10.1186/s13287-020-01919-w)

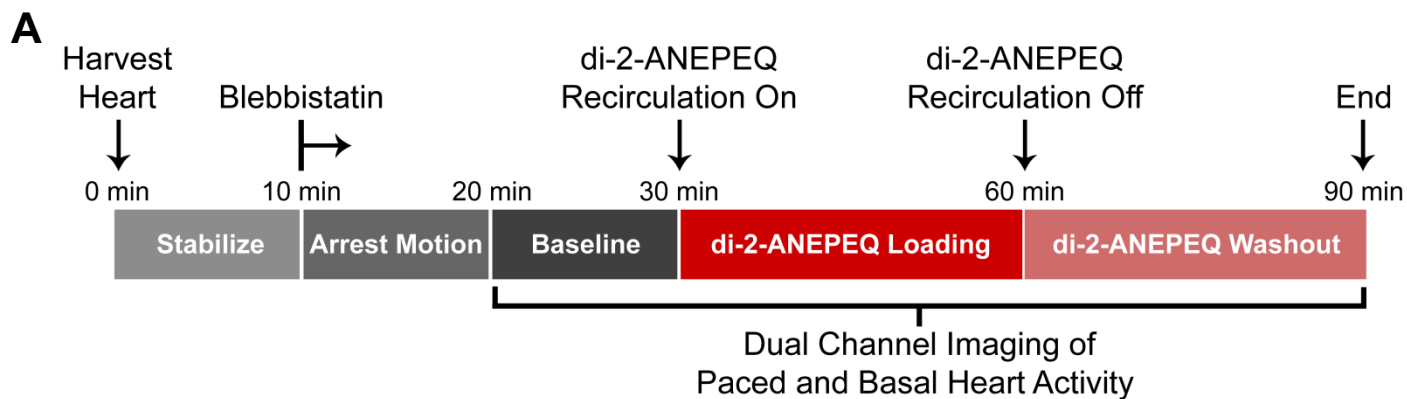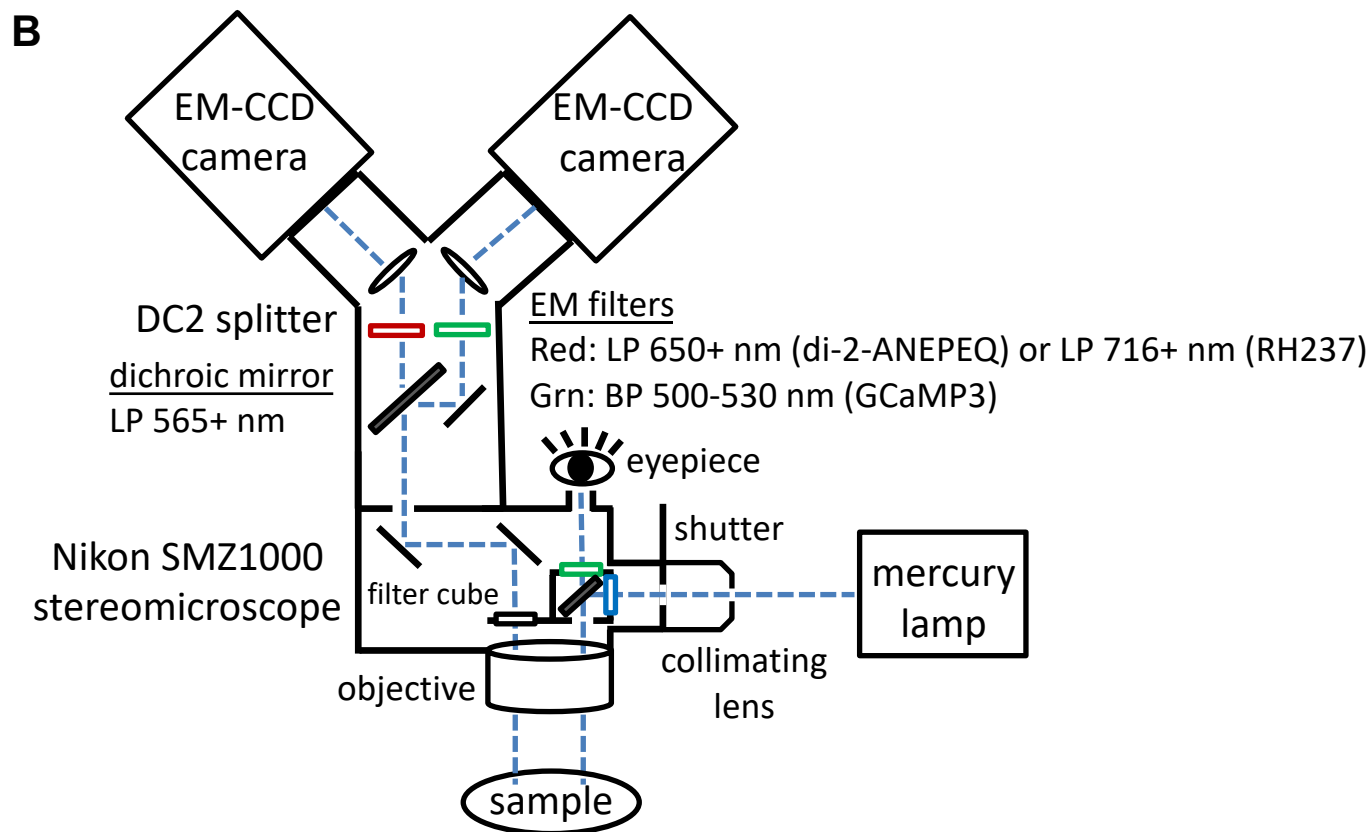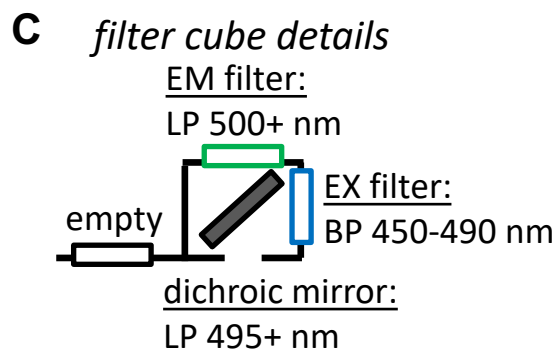

**Fig. S1**

Supplement: Supplementary file 2 — Additional file 1: Supplementary Fig. S1. Experimental imaging protocol and cardiac optical mapping system. A: Overview of typical experimental protocol used to image hearts ex vivo before, during and after perfusion with di-2-ANEPEQ. B&C: Schematic of the dual EM-CCD-based imaging system (B) with details of the filter set used for excitation (C). In brief, excitation light was collimated and bandpass filtered to 450–490 nm before being reflected onto the Langendorff-mounted heart. Emitted light was collected and split into the “green” and “red” channels first by a 565 nm longpass dichroic mirror inside the DC2 dual-channel splitter. The “green” channel (i.e., GCaMP3) signal was then further bandpass filtered to 500–530 nm before being imaged by an EM-CCD camera, while the “red” channel (i.e., voltage dye) signal was longpass filtered at 650+ nm for di-2-ANEPEQ imaging or 716+ nm for RH237 imaging before being imaged on a separate EM-CCD camera operated simultaneously. [file 13287_2020_1919_MOESM1_ESM.pdf]

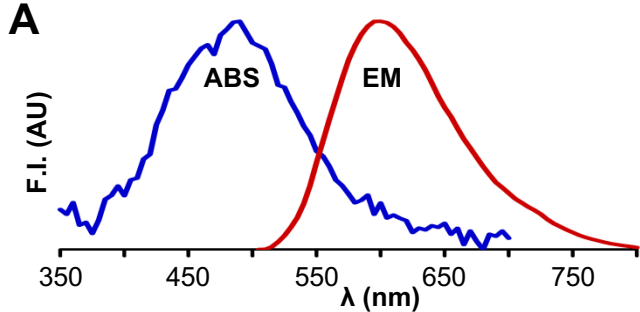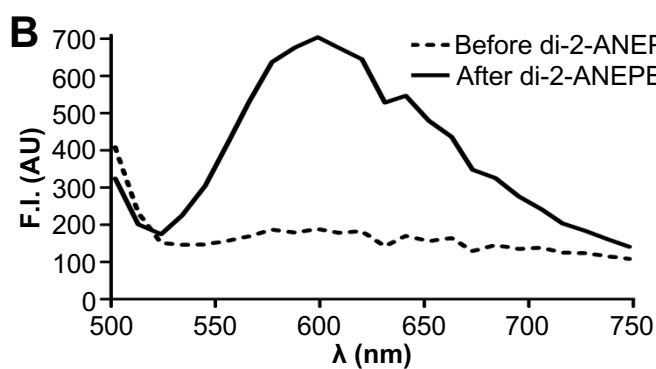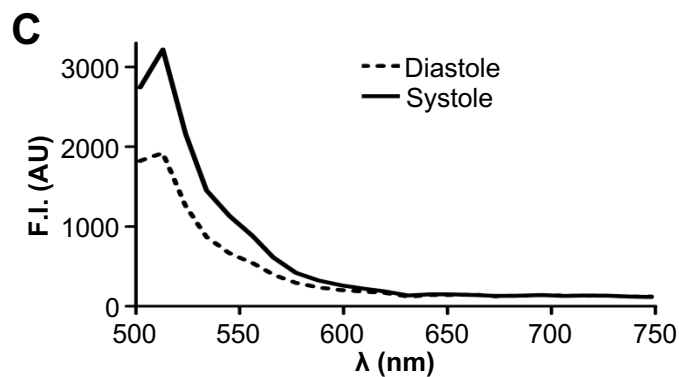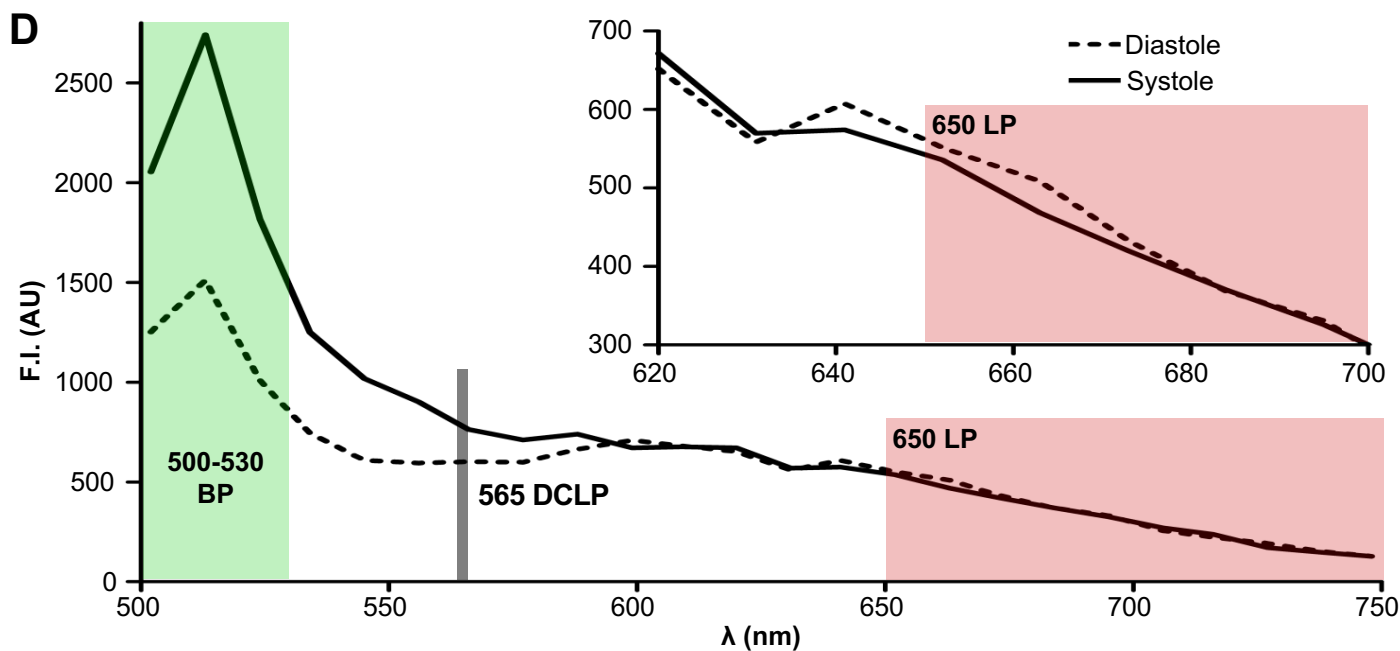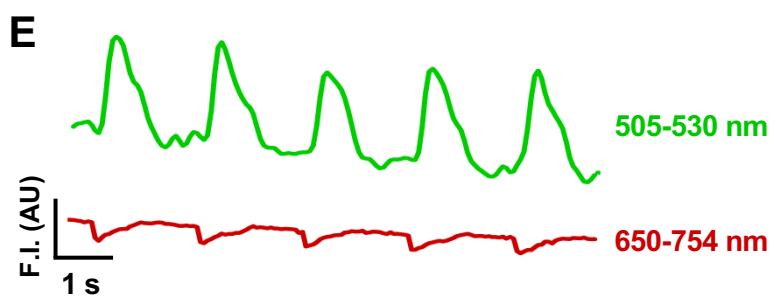

**Fig. S2**

Supplement: Supplementary file 3 — Additional file 2: Supplementary Fig. S2. Spectra of GCaMP3 and di-2-ANEPEQ in hESC-CMs in vitro. A: Absorbance (ABS) and emission (EM) spectra of di-2-ANEPEQ by spectrofluorimetry after loading into hESC-CMs. Note di-2-ANEPEQ-loaded hESC-CMs absorb with a peak at ~ 488 nm and emit with a peak at ~ 600 nm. B: Emission spectra of WT hESC-CMs before and after loading with di-2-ANEPEQ, as determined by confocal lambda scanning. C: Emission spectra of GCaMP3+ hESC-CMs during systole and diastole (solid and dotted traces, respectively). D: Emission of GCaMP3+ hESC-CMs after loading with di-2-ANEPEQ as recorded in both systole and diastole. GCaMP3 mediates a large increase in emission signal during systole, while di-2-ANEPEQ emission exhibits a small spectral shift to the left (best seen in the magnified inset depicting signal at longer wavelengths). The shaded areas denote filter sets selected to separate these fluorophores in all subsequent experiments. E: Mean fluorescence activity over time in a single GCaMP3+ hESC-CM after loading with di-2-ANEPEQ, using the filter sets depicted in panel D. Note that while GCaMP3 fluorescence increases upon depolarization, di-2-ANEPEQ fluorescence decreases at the emission wavelengths selected for detection (> 650 nm). [file 13287_2020_1919_MOESM2_ESM.pdf]

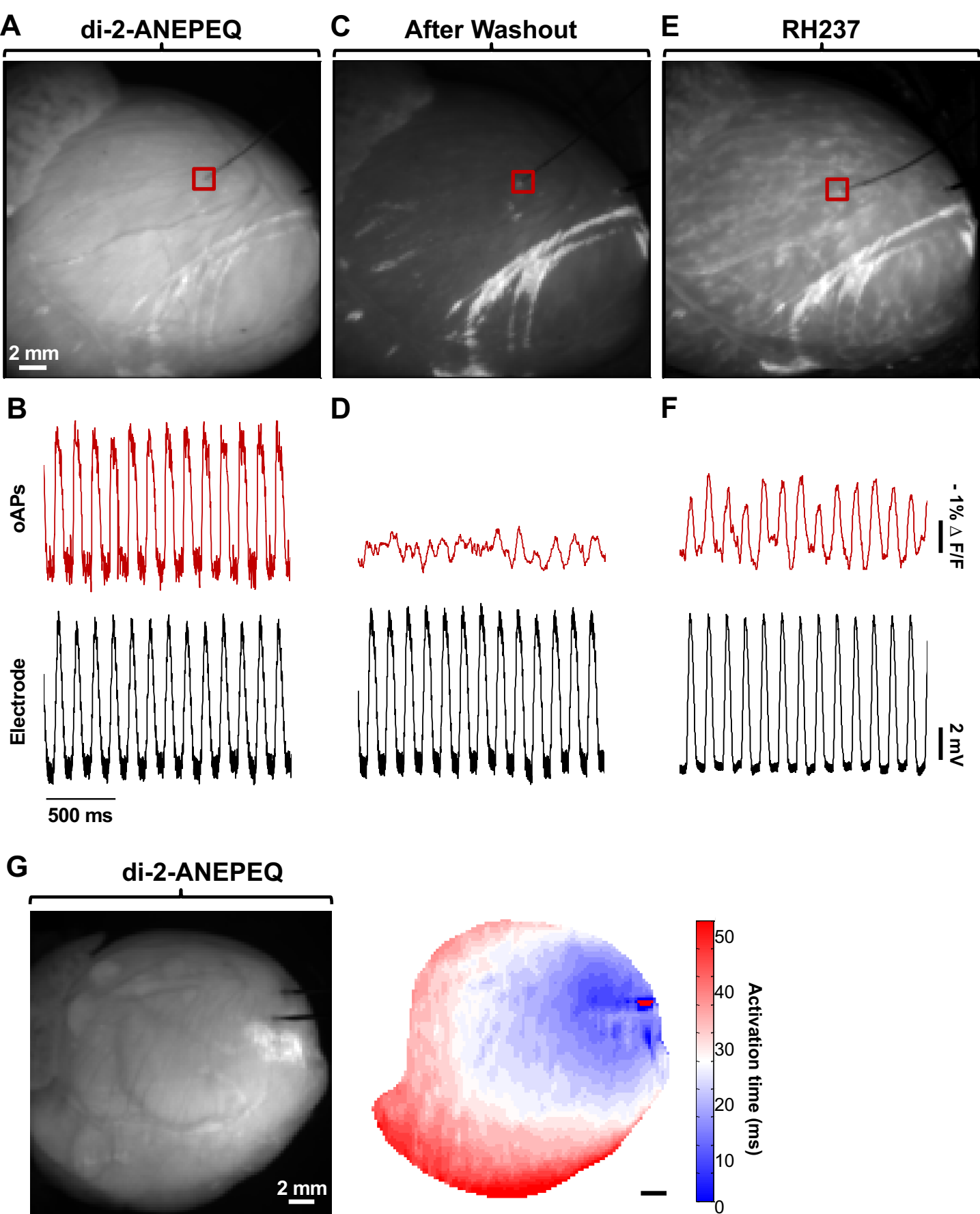

**Fig. S3**

Supplement: Supplementary file 4 — Additional file 3: Supplementary Fig. S3. di-2-ANEPEQ reliably reports myocardial electrical activity. Still images and fluorescence traces on the “red” channel from a naïve heart (uninjured and not transplanted) acquired after di-2-ANEPEQ loading (A&B), after di-2-ANEPEQ washout (C&D), and then after loading with RH237 (E&F). These fluorescent oAPs (red traces) were recorded simultaneously and showed good temporal agreement with direct voltage recordings via sharp electrode (black traces). G. Still image (left) and voltage activation map (right) from a naïve heart. [file 13287_2020_1919_MOESM3_ESM.pdf]

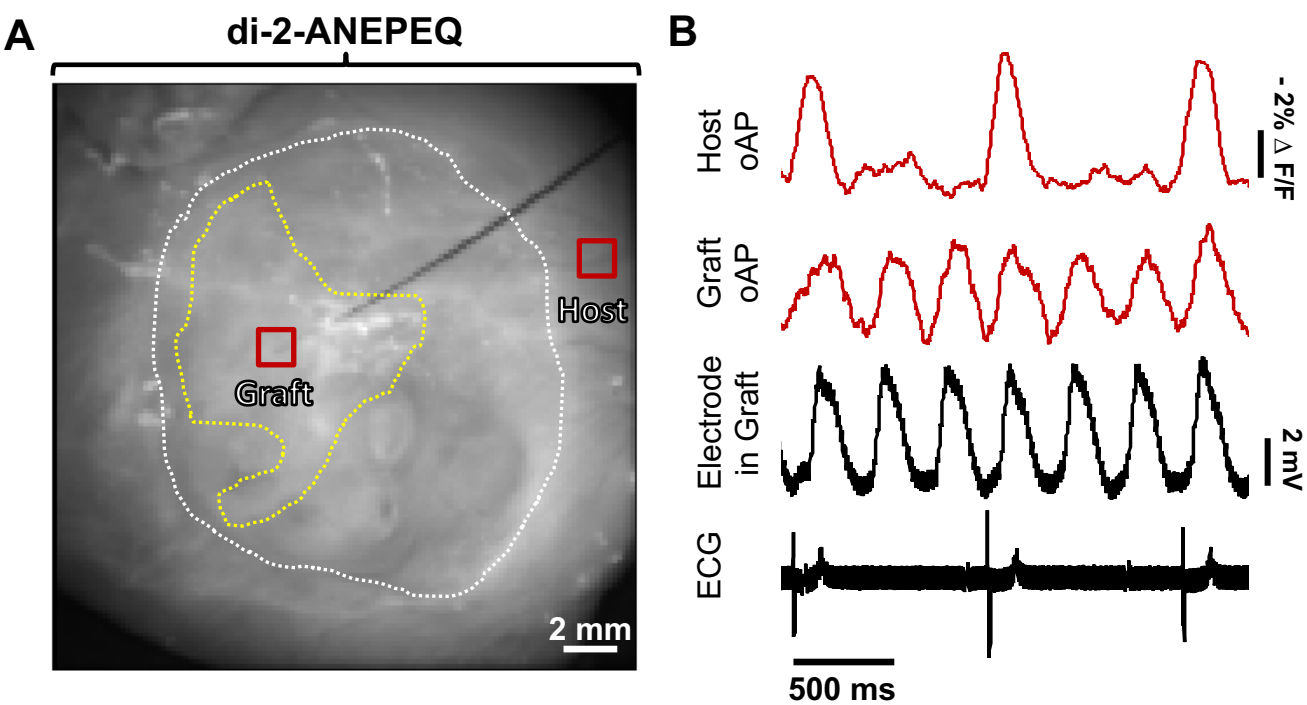

**Fig. S4**

Supplement: Supplementary file 5 — Additional file 4: Supplementary Fig. S4. Simultaneous di-2-ANEPEQ imaging and direct electrode recording of hESC-CM graft electrical activity. A cryoinjured heart with hESC-CM graft (H7 line) was impaled with a sharp electrode within the graft tissue to correlate the di-2-ANEPEQ-derived voltage signal with direct intracellular voltage recordings. A: Epicardial still image taken on the di-2-ANEPEQ channel, showing the uncoupled hESC-CM graft footprint (yellow dotted line) inside the cryoinjury region (white dotted line) and the recording electrode positioned in graft tissue. Two ROIs are indicated: one in host myocardium outside the cryoinjury zone and one in hESC-CM graft tissue overlying the tip of the recording electrode. B: Simultaneously acquired oAPs for these two ROIs, as well as the simultaneously acquired direct voltage and ECG recordings. Note the excellent temporal correlation between the direct electrode recordings and di-2-ANEPEQ-derived oAPs from this hESC-CM graft, which was uncoupled from the host. [file 13287_2020_1919_MOESM4_ESM.pdf]

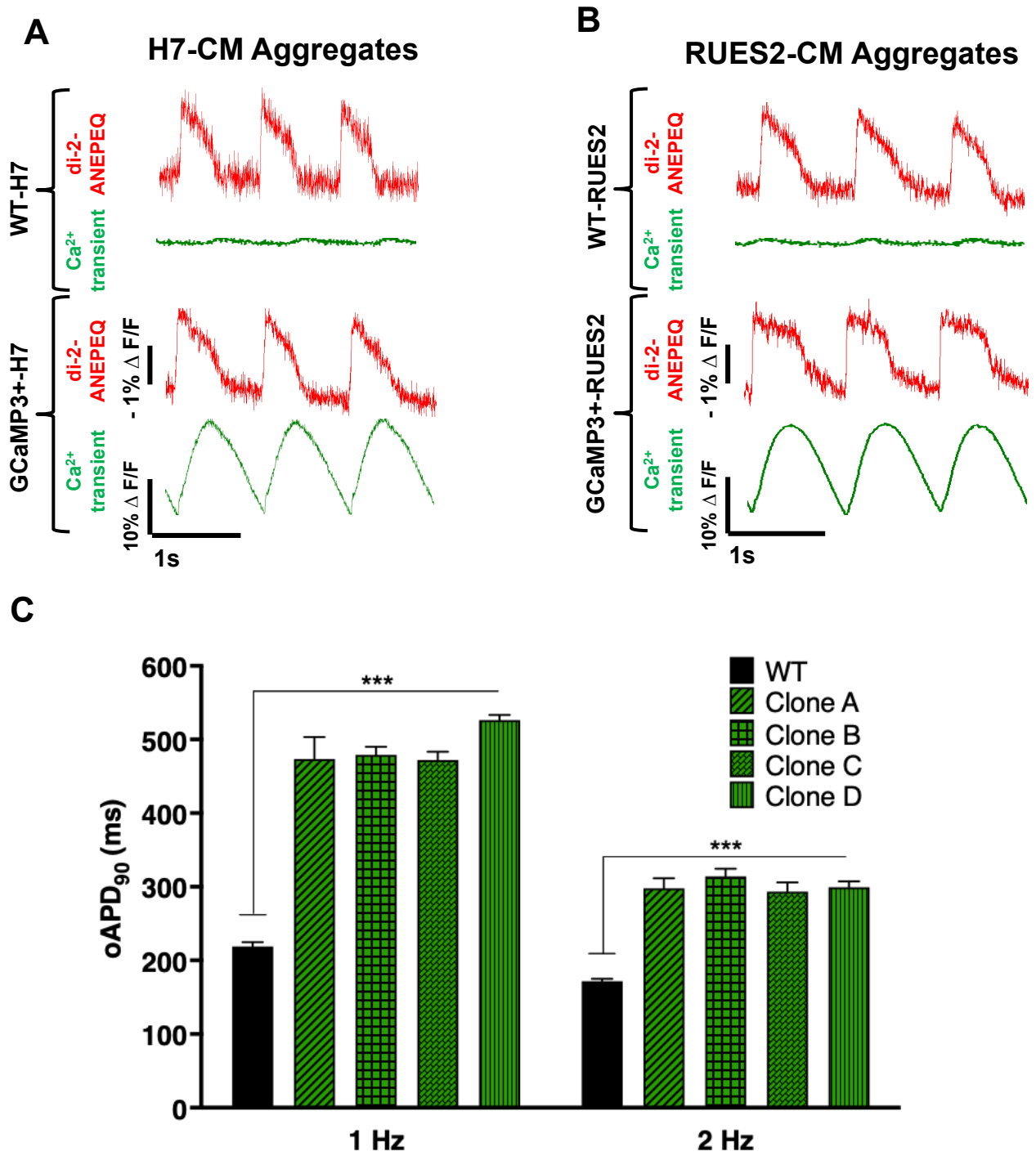

Fig. S5

Supplement: Supplementary file 6 — Additional file 5: Supplementary Fig. S5. In vitro optical action potentials from cardiomyocytes from multiple hESC lines. A, B: Representative fluorescence traces from WT or GCaMP3 cardiomyocyte aggregates generated from either the H7 (A) or RUES2 (B) hESC lines. Aggregates were simultaneously imaged on the di-2-ANEPEQ (“red”, voltage) and GCaMP3 (“green”, intracellular calcium) channels during pacing at 1 Hz. While oAPD was similar between WT and GCaMP3+ H7 hESC-CM aggregates, GCaMP3+ aggregates from the RUES2 line typically showed oAPD prolongation relative to their WT counterparts. C: To rule out the possibility that the apparent AP-prolonging effect of GCaMP3 was just reflective of idiosyncratic hESC clones, oAPs were acquired from hESC-CM aggregates formed from four different GCaMP3+ ESI-17 hESC lines. Cardiomyocytes from all four clones showed similar increases in oAPD relative to WT controls. [file 13287_2020_1919_MOESM5_ESM.pdf]
